# Sleep Modulates the Neural Substrates of Both Spatial and Contextual Memory Consolidation

**DOI:** 10.1371/journal.pone.0002949

**Published:** 2008-08-13

**Authors:** Géraldine Rauchs, Pierre Orban, Christina Schmidt, Geneviève Albouy, Evelyne Balteau, Christian Degueldre, Caroline Schnackers, Virginie Sterpenich, Gilberte Tinguely, André Luxen, Pierre Maquet, Philippe Peigneux

**Affiliations:** 1 Cyclotron Research Center, University of Liège, Liège, Belgium; 2 Inserm-EPHE-Université de Caen Basse-Normandie, Unité de Recherche U923, GIP Cyceron, Caen, France; 3 Functional Neuroimaging Unit, University of Montréal, Montréal, Canada; 4 UR2NF - Neuropsychology and Functional Neuroimaging Research Unit, Université Libre de Bruxelles, Brussels, Belgium; Harvard Medical School, United States of America

## Abstract

It is known that sleep reshapes the neural representations that subtend the memories acquired while navigating in a virtual environment. However, navigation is not process-pure, as manifold learning components contribute to performance, notably the spatial and contextual memory constituents. In this context, it remains unclear whether post-training sleep globally promotes consolidation of all of the memory components embedded in virtual navigation, or rather favors the development of specific representations. Here, we investigated the effect of post-training sleep on the neural substrates of the consolidation of spatial and contextual memories acquired while navigating in a complex 3D, naturalistic virtual town. Using fMRI, we mapped regional cerebral activity during various tasks designed to tap either the spatial or the contextual memory component, or both, 72 h after encoding with or without sleep deprivation during the first post-training night. Behavioral performance was not dependent upon post-training sleep deprivation, neither in a natural setting that engages both spatial and contextual memory processes nor when looking more specifically at each of these memory representations. At the neuronal level however, analyses that focused on contextual memory revealed distinct correlations between performance and neuronal activity in frontal areas associated with recollection processes after post-training sleep, and in the parahippocampal gyrus associated with familiarity processes in sleep-deprived participants. Likewise, efficient spatial memory was associated with posterior cortical activity after sleep whereas it correlated with parahippocampal/medial temporal activity after sleep deprivation. Finally, variations in place-finding efficiency in a natural setting encompassing spatial and contextual elements were associated with caudate activity after post-training sleep, suggesting the automation of navigation. These data indicate that post-training sleep modulates the neural substrates of the consolidation of both the spatial and contextual memories acquired during virtual navigation.

## Introduction

Our understanding of the processes generating and maintaining sleep is rapidly increasing. Amongst the different functions that sleep may fulfill, lines of evidence gathered from cell recordings in rodents to behavioral and neuroimaging data in humans provide support to the idea that sleep participates in the long-term consolidation of recently acquired memories [1]–[4]. In this perspective, consolidation is defined as the process by which new memories are gradually transformed from an initially labile state (in which they are vulnerable to disruption) to a more permanent state in which they are stabilized and/or strengthened [1]. At the system level, consolidation is a prolonged process that involves a gradual reorganization of the brain areas supporting memory retrieval [5].

In the domain of declarative memory, neuroimaging and brain stimulation techniques have provided convincing evidence for sleep-dependent changes in the representation of recently acquired verbal memories [6]–[9]. Human declarative memory is deemed to build upon a system used for spatial learning in animals, dependent upon the hippocampus and medial temporal lobes. In this respect, declarative (or episodic) and spatial memory are closely related domains since they initially rely on the same brain areas. In humans, studies of spatial memory have shown that navigation ability and performance improvement are associated with hippocampal activity both during wakefulness [10] and post-training slow wave sleep [11], suggesting a learning-dependent modulation of hippocampal activity during human sleep that reflects the offline processing of recent memory traces. Additionally, it was shown that learning-related brain activity is restructured during sleep in such a way that spatial navigation in a virtual environment, initially relying on a spatial-based, hippocampus-dependent strategy, becomes progressively contingent on a more automated response-based strategy mediated by the caudate nucleus [12].

However, it must be taken into account that virtual navigation is not a pure process from a neuropsychological standpoint, as it involves many cognitive operations and different memory components, among which the spatial and contextual memory representations. More precisely delineated, a spatial memory representation involves the creation of and/or the access to a cognitive map of the environment where the spatial relationships between the streets are specified independently of the salient features of the environment. For instance, when attempting to reach the church from the hospital, one can keep in mind an “abstract” map-like representation indicating the appropriate direction to follow at each crossroad, independently of specific environmental cues along the way. Besides this “streets configuration” component however, a second, complementary process can be used, which refers to a contextual memory representation (or “landmarks memory”) in which specific associations between salient landmark objects and their milieu are stored. For instance, one may remember that from hospital to school, there is a right turn just after the grocery store and then a left turn in front of the church. Besides prominent commonalities between these two primary components found in a large hippocampo-neocortical network, classically involved in navigation, there is evidence in favor of a partial segregation between their neural substrates. Indeed, we have found that hippocampal activity mostly supports spatial memory processes, whereas activity in parahippocampal, frontal, posterior parietal and lateral temporal cortices supports the contextual memory component [13]. In this context however, it remains unclear whether post-training sleep globally promotes the consolidation of all memory components involved in virtual navigation, or rather reinforces specific aspects of the overall memory representation.

Within an episodic memory task, the spatial and temporal components of verbal memory have been shown to benefit from sleep, whereas the effect of sleep was much less stringent when considering the factual dimension of memories [14]. This suggests that post-training sleep does not necessarily support the consolidation of all the cognitive components engaged in a given task. In the navigation task, one may surmise that the contextual memory component (which may be assessed by means of a task probing associations between landmarks and their milieu) should be particularly sensitive to sleep deprivation. Indeed, context-based memory tasks require creating novel associations between stimuli and their milieu, a binding operation that relies on the hippocampus [15], [16]. Nevertheless, in our previous study [13], we showed that accurate contextual memory performance is also associated with activity in parahippocampal and frontal cortices. Given that part of the memory consolidation process is supposed to occur during post-learning sleep by means of a dialogue between the hippocampus and neocortical areas [17], [18], and that sleep deprivation alters the functioning of frontal lobes [19] involved in contextual memory, it can be hypothesized that sleep deprivation will impede post-learning processes and result in decreased performance on the contextual task. Besides, one can expect a detrimental effect of sleep deprivation on spatial memory processes on the basis of rodent data gathered using complex spatial tasks [1].

**Figure 1:**
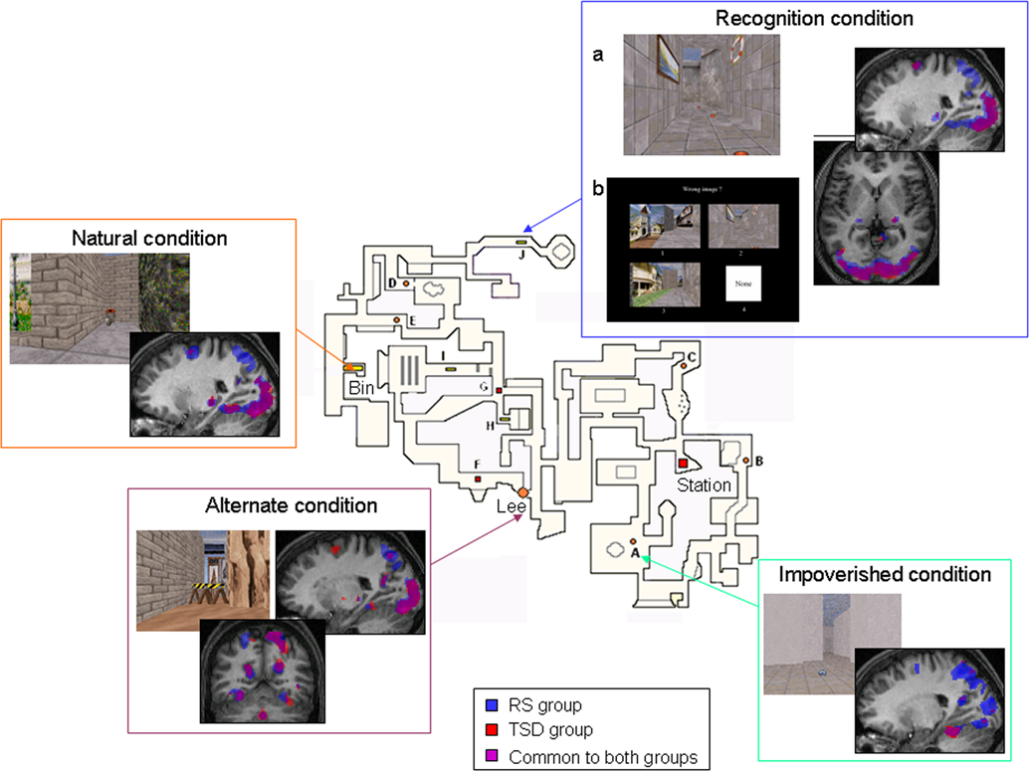
Virtual environment and navigation tasks. The map depicts an aerial view of the colour 3D virtual town in which subjects navigated at the ground level using a keypad. The 10 possible starting points are represented by letters (from A to J) with associated symbols and colours indicating the location to reach, out of 3 possible targets (Bin, Lee and Station). The 4 snapshots display samples of the environment as seen by the participant in the Natural, Recognition, Alternate or Impoverished conditions. For the Recognition task, subjects first navigated in the environment following colour dots on the ground (left panel). They were instructed to determine whether and where environmental changes had been made as compared to the town explored during the training period. At the end of each walk, a four-choice panel composed of 3 pictures taken from the path (one of them representing a change made in the environment), and a white square was presented. Subjects had to respond by selecting the modified image or the white square if they thought that no modification had been made (right panel). Also displayed in the insets is the navigation-related activity in each condition. Each image thus shows areas where BOLD response is greater than the mean (baseline) activity, at the population level, during navigation blocks. Contrasts are displayed at p^corr^ <0.05 (FWE, corrected for multiple comparisons in the whole brain volume) superimposed on a representative subject's MRI, in RS (blue blobs) and TSD participants (red blobs). Purple blobs represent activations observed in both groups. For the Alternate condition, the image represents activations during the whole blocks of navigation, that is not distinguishing between Detour or Routine behaviours.

To delineate further how and to what extent sleep contributes to the consolidation of novel memories after navigation in a virtual town, we have mapped regional cerebral activity using functional magnetic resonance imaging (fMRI) during various tasks specifically designed to tap either the spatial (Impoverished and Alternate conditions) or the contextual memory component (Recognition condition), or both (Natural condition), with or without intervening sleep deprivation after one hour of learning. In the Natural, Impoverished and Alternate conditions, subjects had to retrieve in no more than 35s the route between two locations in the learned environment. In the Impoverished condition, the environment was plainly deprived of any wall/ground feature and objects, promoting the use of spatial representations to successfully perform the task. In the Natural and Alternate conditions, the environment was the same as during the exploration, allowing the use of both contextual and spatial memory representations. In the Alternate condition however, direct pathways between starting and target points were blocked to promote alternative route-finding strategies that rely more on spatial representations (Figure 1). In the Recognition condition, subjects had to pay attention to the environmental features of the town while following dots between two locations, thus the spatial requirements of the task were minimized while the contextual representations were probed. Subjects had then to determine whether environmental changes were made as compared to the exploration by selecting their response on a multiple-choice panel (Figure 1). In all subjects, a one-hour training free exploration period was performed outside of the scanner, and functional MRI data acquisition took place 72 h after encoding. One group was fully deprived of sleep during the first post-training night (total sleep deprivation, TSD), whereas the other was not (regular sleep, RS). All subjects slept normally during the second and third post-encoding nights. Our results indicate that post-training sleep reshapes the neural correlated subtending both the spatial and contextual memory representations in the human brain.

## Results

### Behavioural results

#### Psychomotor Vigilance task (PVT)

In order to rule out the possibility of persisting effects of sleep deprivation (e.g., tiredness, lower vigilance) at retesting, a psychomotor vigilance task (PVT) [20] was administered before learning and testing. Median reaction times did not differed between sessions (day 1 *vs.* day 4) or condition (RS *vs.* TSD) (*ps*>0.47). A similar analysis conducted on the mean of the 10% slowest reaction times failed to reveal any significant effect of group nor interaction between these factors (*ps*>0.1; see Supplemental Information Text S1).

#### Sleep and actigraphic data

Sleep duration and quality were estimated by means of self-reports the nights preceding learning and the fMRI scanning session. Mean sleep duration was not significantly different between groups on both nights. Likewise, subjective sleep quality was equivalent between groups for each night (*ps*>0.34; see Supplemental Information Text S1).

Analyses carried out on actigraphic data showed as expected a larger activity in TSD than RS participants during the first post-training night (p<0.001), whereas no between-group difference was detected for the two recovery nights (*ps*>0.22; see Supplemental Information Text S1). Together with PVT data, these results indicate that testing subjects two nights after a sleep deprivation was a time interval sufficient to dissipate most of the side-effects of sleep deprivation (e.g. tiredness, low vigilance).

#### Navigation memory conditions

Topographical knowledge tested in the Natural condition was equivalent between RS and TSD groups at the end of the learning period (unpaired Student t-test p>0.72, Table 1). Most importantly, performance at delayed testing three days later in the Natural, Impoverished, Alternate and Recognition conditions (Table 1) was also not different between groups allowed to sleep (RS) or not (TSD) on the first post-training night (*ps*>0.78). This result replicates but also extends to more specific memory conditions our prior study disclosing a lack of observable effect of sleep deprivation on navigational behavior [12]. A comparison of place-finding performance at the end of training and at delayed testing revealed a stabilization of performance that was equivalent between groups in the Natural condition (see Table 1).

**Table 1:** Behavioral performance.

			Group		p value*
			RS	TSD	
Performance at the end of the training session			11.3±7.9	12.4±7.8	0.72
*Natural condition*					
		mean performance	11.6±8.1	12±8.7	0.9
		mean effective speed	1.1±0.1	1.2±0.2	0.1
		time spent hesitating	23.3±15	15.3±12.4	0.17
		mean number of hesitations at cross-roads	5.3±3.6	3.2±2.7	0.11
		mean number of dead ends visited	2±1.7	1.5±1.6	0.46
		Change in performance (number of cases) (1)	0.3±6	−0.4±6.2	0.77
		mean performance at 6 months	18.5±10.1	15.3±6.2	0.37
		Change in performance (number of cases) (2)	7.2±5.3	3.3±6.9	0.14
*Impoverished condition*					
		mean performance	24.8±9.6	24.2±9.2	0.87
		mean effective speed	1±0.1	1±0.1	0.38
		time spent hesitating	41.1±17.9	35.1±21.7	0.47
		mean number of hesitations at cross-roads	8.5±2.9	7.2±4.2	0.37
		mean number of dead ends visited	3.3±1.7	3.8±1.9	0.58
		mean performance at 6 months	25.5±6.1	25.9±6.6	0.89
		Change in performance (number of cases) (2)	0.9±9.3	1.7±8.1	0.83
*Alternate condition*					
		mean performance (3)	22.2±8	21.8±6.5	0.91
		mean performance (4)	9±8	9.1±4.9	0.94
		mean effective speed	1.1±0.1	1.1±0.2	0.86
		time spent hesitating	13±9.3	7.3±5.6	0.08
		mean number of hesitations at cross-roads	1.9±1.8	1.7±1.5	0.71
		mean number of dead ends visited	0.7±0.9	1.4±1.6	0.17
		mean time spent having a detour strategy	18.9±4.6	17.4±2.7	0.36
		mean time spent having a routine strategy	9.9±1.6	10.9±2	0.19
		mean time spent being lost	4.5±2.6	6.4±3.8	0.15
		mean performance at 6 months (3)	26±10.3	22.5±6.7	0.35
		mean performance at 6 months (4)	11.2±7.3	9.9±5.1	0.61
		Change in performance (number of cases) (2,4)	2.4±5.7	0.7±6.2	0.51
*Recognition task*					
		mean percentage of correct recognitions	64.2±18.7	62.1±15.4	0.78
		mean reaction times (s)	5.1±2.6	5.1±2.3	0.85
		mean performance at 6 months	58.7±10.3	50±14	0.14
		% change in performance (2)	−15.3±51.3	−42.1±80.5	0.18

For the Natural, Impoverished and Alternate conditions, a positive value indicates a loss of performance. In contrast, for the Recognition condition, a positive value indicates a gain in performance.

(1): as compared to training.

(2): as compared to day 4.

(3): calculated as the distance remaining to the target.

(4): calculated as the distance relative to an imaginary point located 35 units apart the starting point, on the new optimal path.

* : Statistical significance was set at p<0.001 (Bonferroni correction).

More detailed analyses of memory performance, conducted on specific parameters including effective speed, time spent hesitating, making a detour, a routine strategy or being lost, number of hesitations at cross-roads, number of dead ends revisited, and reaction times in Recognition memory condition also failed to disclose any significant between-group difference (.1<*ps*<.94; Table 1).

Literature reports suggest that performance on spatial tasks might differ between men and women [21]. A two-way analysis of variance (ANOVA) conducted on performance obtained at the end of the training session with group (RS *vs.* TSD) and gender (Male *vs.* Female) as between-subjects factors indeed disclosed a main effect of gender (F(1,20) = 10.1, p<0.005), but no group effect (F(1,20) = 0.17, p<0.68) nor interaction between gender and group factors (F(1,20) = 0.03, p = 0.88). Similarly, analyses conducted on delayed performance at day 4 in the Natural, Impoverished, Alternate and Recognition conditions only revealed a main effect of gender in the Natural condition (F(1,20) = 12.88, p = 0.002). Data inspection revealed significantly poorer performance in females than males, but no difference survived Bonferroni correction for multiple comparisons (Supplemental Table S1). No interaction between gender and post-training sleep condition was observed, whatever the task or behavioral measure considered.

Finally, residual knowledge of the environment was further probed after a delay of about 6 months (mean delay±SD: 193.3±23.1 days). The underlying hypothesis was that, even if sleep deprivation has no significant effect on behavioral performance 3 days after learning, the memory trace could be weaker in the TSD group and therefore decay more rapidly in this group as compared to the RS one. Again, statistical analyses carried out on the variables previously used (mean distance, number of correct recognitions …) as well as on estimated loss in performance over 6 months failed to reveal any between-group differences (Table 1).

### Post-training sleep-dependent reorganization of brain activity

A detailed analysis of the cerebral networks specifically engaged during navigation in each of the four experimental conditions has been published elsewhere [13]. We will report here for information only the main effects of navigation in each condition (Figure 1), and focus on the brain areas differentially engaged in these navigation conditions as a function of the availability, or not, of sleep on the first post-training night.

#### Natural condition

In line with previous reports [10]–[13], [22], [23], place finding in a natural condition was associated with increased blood-oxygen level-dependent (BOLD) responses in an extended hippocampo-neocortical network, both in TSD and RS groups (p^FWEcorr^<0.05; Figure 1). Besides activations in the hippocampus, navigation in the Natural condition increased BOLD responses in the superior frontal and precentral gyri, the supplementary motor area and the middle cingulate cortex. Activations were also found in the superior or inferior parietal gyrus, the precuneus, the cerebellum and in the fusiform, lingual and middle occipital gyri (see Supplemental Table S2 for details of navigation-related activations in each group, 72 h after training). Looking at sleep-dependent effects, brain activity was higher in RS than TSD participants in a set of cortical areas including the left supramarginal gyrus, left precentral gyrus, right prefrontal cortex and left fusiform gyrus (Table 2a). The opposite contrast (i.e., testing for higher brain response in TSD than RS subjects) did not yield any significant results (Table 2a). In this latter analysis, posterior probability maps [24] indicated a very low probability to obtain a caudate or hippocampal activation at locations previously reported in the literature (all *P* values <3%).

**Table 2:** 

			MNI coordinates (mm)			
	Comparison	Anatomical region	x	y	z	Z score
**a. Post-training activity during the Natural condition.**						
	*RS>TSD*	L supramarginal gyrus	−46	−32	34	3.86
		L precentral gyrus	−30	−12	58	3.68
		L inferior occipital gyrus	−42	−84	−14	3.60
		L superior frontal gyrus	−30	−2	62	3.25
		R middle frontal gyrus	26	50	−4	3.30
		L fusiform gyrus	−34	−40	−12	3.23
	*TSD>RS*		no suprathreshold clusters			
**b. Positive correlations between brain activity and individual performance in each test of the Natural condition.**						
	*RS>TSD*	L cerebellum	−6	−38	−26	3.97
		L caudate	−10	4	12	3.72 *
		B superior frontal gyrus	2	32	32	3.69
		B cingulum				
		L inferior frontal gyrus	−36	28	22	3.62
		R caudate	14	14	18	3.39 *
		L inferior frontal gyrus	−40	20	−4	3.33
		R middle frontal gyrus	46	38	30	3.32
		L superior medial frontal gyrus	0	60	30	3.31
		L superior medial frontal gyrus	0	62	0	3.30
		R lingual gyrus	24	−46	−4	3.26
		R lingual gyrus	12	−40	−4	3.25
		L middle temporal gyrus	38	32	0	3.20
		L superior temporal gyrus				
	*TSD>RS*		no suprathreshold clusters			

a. Brain areas in which activity during tests of the Natural condition was higher in the RS than in the TSD group (RS>TSD) or higher in the TSD than in the RS group (TSD>RS).

b. Brain areas in which activity was positively correlated with navigation performance (within-subject correlations), and more so in the RS than in the TSD group or conversely.

Coordinates x, y, z (mm) are given in standard stereotactic MNI space. All regions listed are statistically significant at the p<0.001 level, excepted ^*^: psvc(10 mm)<0.05, significant after correction in a small spherical volume (radius 10 mm) around navigation-related coordinates previously reported in the literature (see Supporting Information Text S1). For brevity, each region is listed only once; when several peaks were observed in the same region, the coordinates refer to the strongest activation. L: left; R: right.

**Figure 2:**
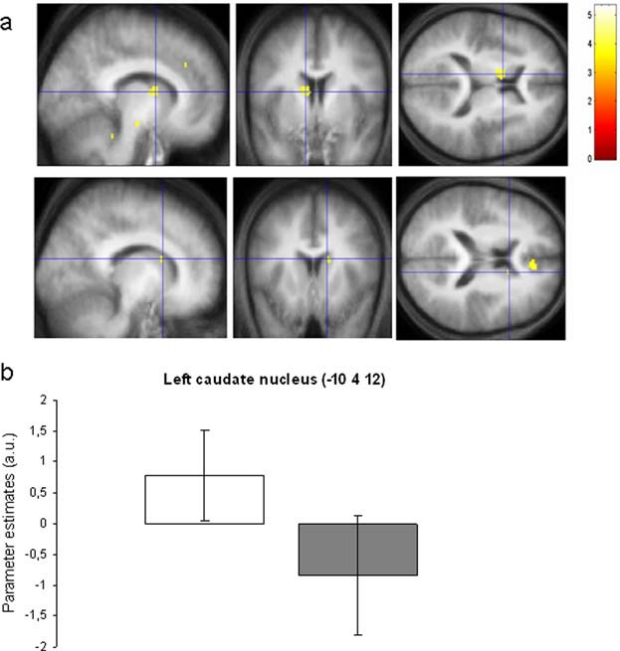
Within-subjects correlation analyses between brain activity and trial to trial performance in the Natural condition. Contrasts are displayed at p<0.001 (uncorrected) superimposed on the average T1-weighted MR scan. a) Higher correlations with performance in the RS than TSD group in the left (−10, 4, 12; Z = 3.72; top panel) and right (14, 14, 18; Z = 3.39; bottom panel) caudate nuclei. b) Parameter estimates (arbitrary units±standard deviations) of correlation between performance and brain activity in the left caudate nucleus (−10 14 12), indicating a positive association in the RS group (white plot), and a negative association in the TSD group (grey plot).

Although average activity was similar in both groups in the striatum, correlation analyses revealed that the association between brain activity (main effect of navigation) and intra-individual variations in navigation performance was different between RS and TSD groups in the left (−10 4 12, Z = 3.72) and right (14 14 18, Z = 3.39) caudate nuclei (p^svc(10 mm)^ <0.05; Figure 2) as well as in several cortical areas (Table 2b). Data plots indicate that brain activity in the RS group was positively correlated with performance in both caudate nuclei (Figure 2). No areas were found with higher correlation in the TSD than the RS group. These data confirm prior reports [12], [25] and are in agreement with the hypothesis that navigation efficiency has become more dependent on automated processes in RS than TSD subjects, a behavior thought to be supported by striatal areas.

#### Impoverished condition

Navigation in the Impoverished condition elicited increases in BOLD responses in an extended hippocampo-parahippocampo-neocortical network both in RS and TSD groups (Figure 1; Supplemental Table S3). Besides activations in the hippocampus or in the parahippocampal gyrus, this condition elicited increased brain responses in the superior parietal gyrus, the cuneus/precuneus, the middle occipital and fusiform gyri and the cerebellum. Brain activity was higher during place finding in RS than TSD participants in a set of posterior cortical areas including the left fusiform and lingual gyri, the right inferior parietal gyrus the right calcarine region, as well as the right middle and inferior occipital gyri (Table 3a). The opposite contrast disclosed higher brain response in TSD than RS subjects bilaterally in the inferior frontal gyrus, the left uncus (parahippocampal gyrus) and in the middle temporal gyrus (Table 3a).

**Table 3:** 

			MNI coordinates (mm)			
	Comparison	Anatomical region	x	y	z	Z score
**a. Post-training activity during the Impoverished condition.**						
	*RS>TSD*	L fusiform gyrus	−28	−64	−12	3.48
		R calcarine	18	−86	6	3.42
		R inferior parietal gyrus	30	−48	52	3.32
		R inferior occipital gyrus	46	−68	−16	3.25
		L lingual gyrus	−18	−80	−10	3.23
		R middle occipital gyrus	32	−84	8	3.19 *
	*TSD>RS*	L inferior frontal gyrus	−50	36	−6	3.74
		R inferior frontal gyrus	46	30	−10	3.55
		L uncus	−22	−2	−36	3.43
		L middle temporal gyrus	−52	−36	−6	3.3
**b. Positive correlations between brain activity and individual performance in each test of the Impoverished condition.**						
	*RS>TSD*	R post-central gyrus	44	−20	54	3.39
	*TSD>RS*	L middle temporal gyrus	−42	−60	−4	3.34
		L middle occipital gyrus	−36	−70	16	3.3

**a.** Brain areas in which activity during tests of the Impoverished condition was higher in the RS than in the TSD group or higher in the TSD than in the RS group.

**b.** Brain areas in which activity was positively correlated with navigation performance (within-subject correlations), and more so in the RS than in the TSD group or conversely.

All regions listed are statistically significant at the p<0.001 level, excepted ^*^: p ^svc(10mm)^<0.05.

Correlation analyses revealed a positive correlation between cerebral activity and intra-individual variations in navigation performance that was higher in RS than TSD subjects in the right post-central gyrus (Table 3b). The reverse contrast evidenced higher correlations in the left middle occipital (positive correlation in both groups) and temporal (negative correlation in the RS group; positive in the TSD group) gyri in TSD than RS subjects (Table 3b).

#### Recognition condition

In both groups, navigating to determine whether changes in the environment were made as compared to the learning period elicited increased brain activity in a large neural network including frontal, occipital and posterior parietal areas (Figure 1; Supplemental Table S4). Interaction analyses revealed that brain activity during the navigation task was higher in RS than TSD participants in frontal (left precentral, superior and middle frontal gyri) and occipital areas (left calcarine region, inferior occipital gyri bilaterally and right middle occipital gyrus) as well as in the fusiform gyrus bilaterally (Table 4a). The opposite contrast (i.e., testing for higher brain response in TSD than RS subjects) did not yield any significant results (Table 4a).

**Table 4:** 

			MNI coordinates (mm)			
	Comparison	Anatomical region	x	y	z	Z
**a. Post-training activity during the Recognition condition.**						
	*RS>TSD*	L superior medial frontal gyrus	−10	44	25	4.42
		L fusiform gyrus	−32	−38	−20	4.42
		R inferior occipital gyrus	46	−68	−14	4.11
		L calcarine region	−24	−64	8	3.78
		L inferior occipital gyrus	−42	−84	−16	3.67
		L superior frontal gyrus	−20	50	16	3.50
		L middle frontal gyrus	−26	44	−4	3.43
		R fusiform gyrus	32	−42	−20	3.38
		L precentral gyrus	−42	−4	22	3.24
		R middle occipital gyrus	34	−84	8	3.23
	*TSD>RS*		no suprathreshold clusters			
**b. Positive correlations between brain activity and individual performance in each test of the Recogntion condition.**						
	*RS>TSD*	R superior medial frontal gyrus	12	30	58	3.23
		L superior frontal gyrus	−14	52	40	3.19
	*TSD>RS*	R parahippocampal gyrus	18	−18	−26	3.38

**a.** Brain areas in which activity during tests of the Recognition condition was higher in the RS than in the TSD group or higher in the TSD than in the RS group.

**b.** Brain areas in which activity was positively correlated with navigation performance, and more so in the RS than in the TSD group (top part of the table) or conversely.

All regions listed are statistically significant at p<0.001.

**Figure 3:**
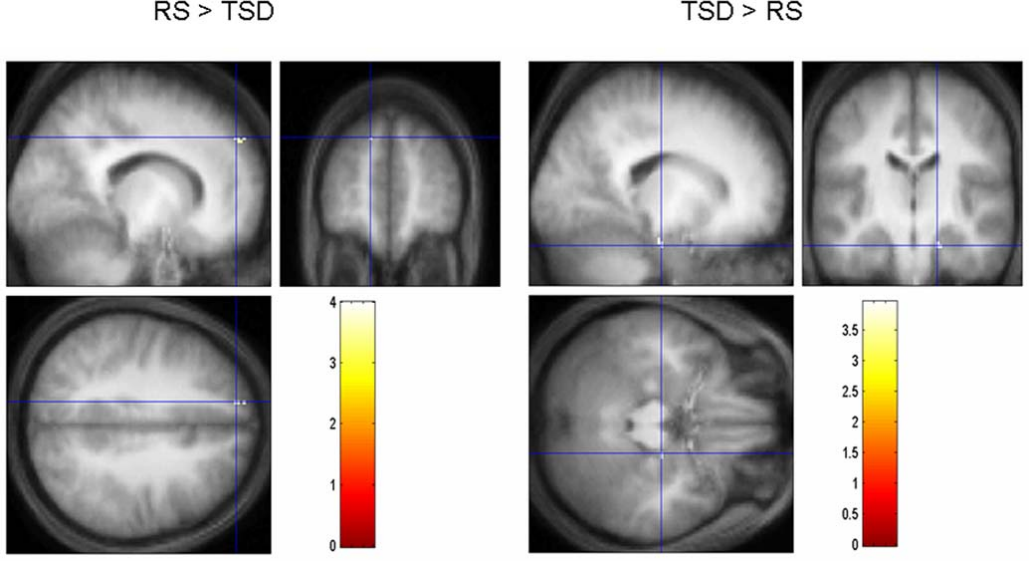
Sleep-dependent modulation of correlation between brain activity and performance in the Recognition condition. Contrasts are displayed at p<0.001 (uncorrected) superimposed on the average T1-weighted MR scan. Correlations were computed at the within-subject level (i.e., between brain activity and individual variations in trial-to trial performance). Left panel: higher correlations in the RS than in the TSD group in the left frontal gyrus (−14 52 40, Z = 3.19). Right panel: higher correlations in the TSD than in the RS group in the right parahippocampal gyrus (18 −18 −26, Z = 3.38).

Analyses revealed higher correlations between correct recognitions and brain activity in the superior frontal gyrus in RS as compared to TSD subjects (Table 4b; Figure 3). The reverse contrast evidenced higher correlation in the right parahippocampal gyrus in TSD than RS subjects (negative correlation in the RS group, positive correlation in the TSD group; Table 4b; Figure 3).

#### Alternate condition

In both groups, finding an alternate route to the target (i.e., detour strategy) elicited activations in the parahippocampal gyrus as well as in frontal, occipital and posterior parietal cortices (Figure 1), but also, more surprisingly, in the caudate nucleus previously associated with automation of navigation [12], [25]. A very close network emerged when analyzing periods during which subjects were walking along a well-known route, i.e., having a routine strategy (Supplemental Table S5). Interaction analyses disclosed higher activation during detours in the right cuneus (20 −82 8, Z = 3.25, p<0.001) in RS than TSD subjects. The reverse contrast revealed higher activity in TSD than RS participants in the right superior temporal gyrus, the anterior cingulate cortex and the right gyrus rectus (Table 5). As for the routine strategy, subjects in the RS group exhibited more activity in the left parahippocampal gyrus (−34 −44 −10, Z = 3.19, p^svc(10mm)^<0.05) and the right precuneus (16 −66 24, Z = 3.19, p<0.001). The opposite contrast disclosed higher activity in TSD than RS subjects in frontal, temporal and occipital areas (Table 5).

**Table 5:** Post-training activity during the Alternate condition.

			MNI coordinates (mm)			
		Anatomical region	x	y	z	Z score
Main effect of “DETOUR”						
	RS>TSD					
		R cuneus	20	−82	8	3.25
	TSD>RS					
		R superior temporal gyrus	36	−30	8	3.60
		R superior frontal gyrus	24	30	14	3.38
		L anterior cingulate gyrus	−16	10	32	3.33
		R gyrus rectus	2	38	−22	3.22
Main effect of “ROUTINE”						
	RS>TSD					
		L parahippocampal gyrus	−34	−44	−10	3.19 *
		R precuneus	16	−66	24	3.19
		R precuneus	22	−74	40	3.12
	TSD>RS					
		Insula	−36	−24	−4	4.59
		L middle temporal gyrus	48	−16	−16	4.08
		L middle temporal gyrus	−58	−20	−8	3.77
		R middle temporal gyrus	68	−40	−6	3.70
		L precuneus	−16	−54	36	3.67
		L middle temporal gyrus	−46	−74	10	3.48
		L anterior cingulate cortex	−16	10	30	3.39
		L middle temporal gyrus	−56	2	−18	3.39
		R middle cingulate cortex	2	−20	38	3.29
		L superior medial frontal gyrus	−12	56	16	3.24
		L Heschl gyrus	−34	−32	12	3.22

Brain areas in which activity during tests of the Detour or Routine part of the road was higher in the RS than in the TSD group (top part of the table) or higher in the TSD than in the RS group (bottom part). All regions listed are statistically significant at the p<0.001 level, excepted ^*^: p^svc(10mm)^<0.05.

Additionally, a two-way interaction analysis revealed higher activity in the Detour than the Routine parts of the route, and more so in RS than TSD subjects, in the right superior temporal ([62 −28 14], Z = 3.9, p<0.001) and precentral gyri ([52 −8 56], Z = 3.92, p<0.001) as well as in the left middle temporal gyrus ([−50 −38 4], Z = 3.18, p<0.001). Conversely, there was higher activity in the Detour than the Routine condition in the left middle frontal gyrus ([−48 48 −10], Z = 3.38, p<0.001) in TSD than RS subjects. No correlation analysis was performed for this condition since it was not possible to compute a measure of memory performance for only the detour or the routine part of the route.

To summarize our main findings, navigation was supported in all conditions by neural activity in an extended network mostly including hippocampal and parahippocampal areas, as well as frontal, posterior parietal, and occipital cortices. Beyond these commonalities, specific areas of activation were identified in RS relative to TSD participants in the occipital cortex and the fusiform, lingual or parahippocampal gyrus for each of the four conditions. RS participants also activated more frontal areas (precentral, superior and middle frontal gyri) in the Recognition and Alternate conditions. Higher activity in the inferior parietal cortex was also observed in these subjects in the Impoverished condition. Supplementary activation patterns were also observed in TSD relative to RS subjects in the Alternate and Impoverished conditions. These activations are located in the anterior cingulate, frontal and temporal cortices (in the lateral, medial and posterior parts of the temporal cortex). No additional activation was found for TSD subjects in the Natural and Recognition tasks. Notwithstanding, regression analyses revealed higher correlation between parahipppocampal activity and Recognition performance in TSD than RS subjects.

## Discussion

In this study, we aimed at investigating the role of sleep for the post-training processing and consolidation of the spatial and contextual memory components engaged in virtual navigation. In line with prior data obtained in a compound condition that does not allow segregating the respective contribution of memory components [12], our results indicate that sleep during the first night after learning to navigate in a novel virtual environment influences the fate of the newly created memory representations but not their behavioural expression. Most importantly, we have shown here that sleep induces a qualitative reorganization of the brain activity that underlies performance on distinct cognitive components of navigation. We will first discuss the behavioural results and then turn toward the reorganization of brain activity after sleep.

### Behavioural results

Whether allowed to sleep or not on the post-training night, participants did not differ in memory performance at the end of training on day 1, allowing unbiased comparison of navigation performance at delayed testing. When retested 3 days later in the Natural condition, all subjects exhibited satisfying knowledge of the environment as attested by the reduced distance remaining to target (see Table 1). However, no behavioural difference between RS and TSD participants was evidenced, even when using more subtle measures of performance. These findings replicate a prior study conducted using the same Natural condition [12]. Additionally, we show here that our prior finding that sleep deprivation on the first post-training night does not alter subjects' ability to find their way in the virtual town three days later was not due to a limited amount of training in the Orban et al. study [12]. Indeed, subjects were given here twice more practice, and still did not differ after sleep manipulation despite better average performance at delayed testing.

Likewise, navigation performance in the Impoverished condition was not different between the RS and TSD groups. Admittedly, this task was much more difficult than the three others as indexed by lower performance levels [13]. Therefore, difficulty may have prevented automation in navigation within this environment deprived of all its salient landmarks, at least in the present conditions of limited training time (one-hour exposition). This makes more unlikely to disclose sleep-related behavioural differences, if any, in this task thought to test for the spatial component of navigation. Finally, performance was equivalently above chance level in the Recognition condition in RS and TSD subjects, and did not differ either between groups in the Alternate condition. An analysis based on individual navigation behaviour during each test (detour or routine strategy) similarly failed to reveal any significant difference between both groups.

Confronted with such behavioural similarities between the experimental groups, one may surmise that sleep deprivation effects are to be found after a longer delay than 3 days. In this perspective, sleep deprivation may weaken the memory trace which would decay more quickly in the TSD group. To test this hypothesis, we have assessed residual knowledge of the environment in our subjects after a delay of about 6 months. Again, data indicate that although performance logically declined in all conditions, performance levels were similar between groups while remaining above chance level (see Table 1).

Even when dissecting precisely subjects' behaviour in thorough analyses, no significant behavioural difference emerged from the comparison between RS and TSD participants. As in our previous study [12], this lack of behavioural effect stands in sharp contrast with an obvious reorganization of the brain activity underlying retrieval performance, which will be discussed in the next section. These results do not concur with those reported by Peigneux et al. [11] who found a significant overnight improvement of performance. However, in this latter study, subjects received very intensive training (up to 4 hours) in a virtual environment that was more complex and extended in space than the present one. It is possible that the training period administered here was not long enough to favour such an enhancement in performance. Still, one may notice than in the Orban et al. study [12], subjects were trained during 30 minutes (i.e., half of the time spent in the present study) and consistently had a weaker place-finding performance than in our study, indicating that subjects learned to find their way in the virtual town at a level proportional to the exposure time. Thus, it cannot be argued that the lack of sleep-dependent consolidation is merely due to a lack of initial training. Our results also stand in contrast with some data disclosing a benefit of sleep for the consolidation of verbal material in the same memory domain, i.e. declarative memory [26]–[28]. However, if overnight changes in memory performance have been reported in the domain of motor learning [29]–[32], a close inspection of the declarative memory literature indicates that overnight improvements are not the general rule. Indeed, studies most often have reported less decline in performance after a night of sleep than after an episode of wakefulness [6], stabilisation of performance during sleep [33], or equivalent performance between sleep-allowed and sleep deprived subjects at delayed retesting, despite changes in the underlying brain activity (e.g. [7] for learning of emotionally negative stimuli; [12]). Also, another possible explanation for the absence of behavioural effect may be that subjects were tested on the next day in the Peigneux et al. study [11], whereas testing occurred 3 days later in the current study, likewise in Orban et al. [12], due to the need for TSD subjects to recover from sleep deprivation before testing. This may have leaved open the door for alternative processes of consolidation to take place, either with the passage of time or during the two recovery nights. We can also surmise that the restructuring of brain activity during sleep strengthens the memory traces by establishing or reinforcing hippocampo-cortical or cortico-cortical connexions and, therefore, mainly makes memories more resistant to interference [34]. In this perspective, it is possible that a behavioural effect of sleep manipulation would have been seen if our subjects had been exposed to a novel, interfering environment just before retesting. Finally, another plausible explanation may consider the ecological validity of our navigation task, as compared to the material commonly used in the verbal domain, where lists of related or unrelated word pairs are presented to the subject. From this perspective, our navigation task presents several ecologically relevant features since subjects have to navigate from an egocentric perspective in the virtual town, in which they are exposed all along to an environment rich in complexity, details and visual stimulations. As navigation is an everyday behaviour of great functional significance for humans and most animal species, it is also possible that alternate mechanisms are at work that assure the encoding of these complex memories and their access on the long term even when consolidation was sub-optimal, as suggested by differential patterns of brain activity. This interpretation is strengthened by the fact that a similar lack of sleep-related behavioural differences, despite differential brain activity, has been found for the consolidation of emotional memories, which are important in evolutionary terms [7].

### Post-training sleep-dependent reorganization of navigation-related brain activity

Despite an absence of overt behavioural effects, fMRI data analysis revealed different patterns of navigation-related brain activity in RS and TSD participants. At the within-subject level, variations in place-finding efficiency in the Natural condition correlated more with caudate activity in RS than in TSD participants. In line with prior results [12] and with conclusions of the Iaria et al. study [25] in which transfer towards caudate activity was associated with automation, it suggests that subjects who slept on the post-training night may have automated their navigation behaviour. Higher correlations in RS than in TSD subjects were also found in frontal areas known to be involved in memory recollection [35], [36], and in the lateral temporal cortex (superior and middle temporal gyri) engaged in recognition memory [37]. The reverse contrast failed to reveal better integration of behaviour with regional brain activity in TSD than RS subjects, suggesting that navigation after a night of sleep deprivation may be supported by manifold processes, more than by a purely spatial, hippocampus-dependent, or response-based, caudate-dependent, strategy.

Interaction analyses revealed that RS subjects recruited more posterior parietal and occipital areas in the Impoverished condition than TSD participants, whereas the reverse contrast mainly revealed higher involvement of frontal areas in the TSD group. The inferior parietal and middle occipital gyri are known to participate in the fine-grained analysis of complex visuospatial information [38], as well as extrastriate visual areas are known to be involved in mental imagery and retrieval of visual details in memory tasks [39]. It would therefore be tempting to suggest that the higher involvement of occipital areas in RS subjects reflects the fact that they stored a more fine-grained representation of the environment, noticeably including the shape of rooms and corridors, in support to find their way. This hypothesis appears unlikely however. Indeed, not only behavioral performance data but also a qualitative analysis of the post-experimental questionnaire investigating subjects' strategies failed to reveal any significant difference between groups. Thus, 75% of the subjects in both groups reported having used a spatial strategy (e.g., recognition of the shape of rooms, memorization of junctions) at some points during the test. Rather, an alternative hypothesis may be that the RS subjects have activated regions involved in lower-level cognitive processes than TSD participants (occipital vs frontal areas).

In the Alternate condition, data were analyzed taking into account the subject's behaviour all along its way. For each subject, we distinguished between parts of the route during which she/he was using a “detour” strategy to bypass the barriers using less familiar ways to reach the target, as opposed to parts of the route during which she/he was following a well-known path, i.e. a routine strategy. The underlying hypothesis was that subjects who had slept on the post-training night would recruit more strongly the hippocampal/parahippocampal regions to fulfill the increased spatial requirements in the detour part of the road than the sleep-deprived subjects. In contrast, for the routine part of the route, we expected to find a pattern of brain activity close to the one observed in the Natural condition with noticeably activations in the striatum, which has been shown to be involved in the automation of navigation behaviour [25]. An analysis based on detour data revealed higher activity in RS than TSD subjects in the cuneus, known to be involved in the generation of mental images [40] and in spatial-associative strategies during memory retrieval [41]. However, one should be cautious in interpreting this result, since half of our subjects actually claimed that they knew the alternative route to reach the target. It therefore suggests that our distinction between detour and routine behaviours may have been less stringent than expected. Finally, a two-way interaction between behaviour (Detour *vs.* Routine) and post-training sleep condition (RS *vs.* TSD) revealed higher activity in the Detour than in the Routine parts of the route, and more so in RS than TSD participants, in frontal and lateral temporal cortices. It suggests that subjects who slept during the post-training night have engaged more cerebral areas known for their contribution in active retrieval [42] and recognition processes [37] in goal-oriented navigation than sleep-deprived subjects. The reverse contrast disclosed higher activity in TSD than RS subjects in frontal areas, noticeably in the anterior cingulate cortex. Activation in this region has been previously reported in situations where subjects were spontaneously planning a route or were facing unexpected violations in the environment [43].

As for the Recognition task, performance-related analyses revealed higher correlations in RS than TSD subjects in the frontal cortex, and in the parahippocampal gyrus in TSD than RS participants. These results are reminiscent of cerebral patterns associated with recollection and familiarity processes, respectively. Indeed, numerous studies carried out using various methodologies (e.g., Remember/Know paradigm: [44], [45]; event-related potentials: [35]) and in various populations have shown that successful recollection is associated, amongst other brain areas, with activity in frontal regions [36], whereas familiarity mostly relies on the parahippocampal gyrus [46]. Remembering is also associated with activity in “content” regions that are specialized in processing particular types of information and that may subserve the recollection of phenomenological details (mental images, thoughts, …) related to a specific learning episode [47], [48]. Such “content-specific” areas have been identified in studies unravelling the neuroanatomical correlates of recollection, among which extrastriate visual areas (and noticeably the anterior fusiform gyrus), also found in the present study. Because activation of these areas has been associated both with perception [49] and retrieval [50] of visual objects, extrastriate areas might therefore be a storage site for the memories encoded during exploration of the environment.

May differences in cerebral activity observed at delayed testing between RS and TSD groups be the mere consequence of incomplete recovery from sleep deprivation? In line with this proposal, Wu et al. [19] reported that recovery sleep does not fully reverse the decrease in metabolism observed in several brain areas after sleep deprivation. However, those results were obtained after only one recovery night in subjects sleep-deprived for about 30 hours. In the present study, TSD subjects were tested after an additional recovery night and both psychomotor vigilance task results [20] and self-reports failed to evidence any difference between RS and TSD participants in terms of tiredness and vigilance. Additionally, others have suggested that the decline in brain metabolism after sleep deprivation may be less important for complex tasks involving greater cerebral compensation than simple tasks [51].

Another possible explanation for differential patterns of cerebral activity between groups during retrieval, despite a lack of behavioural difference, would be that cerebral activation patterns have changed in the TSD group as a result of wake-dependent shift in the memory representation. For example, it was reported that the movement-based component of a motor skill memory may be preferentially enhanced over wake, whereas the goal-based component enhances over sleep [52]. However, this explanation raises the problem that “extended wakefulness” hardly differentiates from “sleep deprivation”, given that sleep deprivation logically occurs only when the normal duration of wakefulness has been extended. In this respect, we believe that our data yield evidence for an effect of lack of sleep on brain activity. It is also likely that circadian factors may be of importance in the process of memory consolidation. Indeed, some studies have reported that lack of nocturnal sleep is more detrimental than lack of diurnal sleep for memory consolidation [29]. This issue should be tested in dedicated studies however, as our behavioural data do not provide evidence that consolidation in the TSD group was sub-optimal, although qualitatively different in the cerebral networks that subtend spatial and/or contextual memories.

Finally, consolidation of declarative memories most probably takes place at various levels. Besides changes in synaptic strength suggested as the cellular level [53], a dialogue between the hippocampus and the neocortex is established whereby memories will be durably stored, preferentially during sleep periods when the hippocampus projects information towards the neocortex [17], [18]. Several studies have provided evidence for a progressive transfer of novel memories towards neocortical sites, which is accompanied by a disengagement of the hippocampus [54], [55]. The effect is more prominent in subjects allowed to sleep during the post-training night than in sleep-deprived subjects [6], [7]. The present study was not designed to reveal a disengagement of the hippocampal formation, so subjects were not scanned during the learning session. Still, a decrease in hippocampal activity over four days in the Natural condition has previously been reported for both the RS and TSD conditions [12].

### Conclusion

Our data suggest that sleep favors a global, qualitative reorganization of both the spatial and contextual components of recently acquired navigation memories. Experimental evidence from the Natural, compound condition, reinforces the hypothesis that brain activity is restructured during sleep in such a way that navigation known to initially rely on a hippocampus-dependent spatial strategy [25] becomes progressively contingent on a response-based strategy mediated by the caudate nucleus [12], [25], [56]. Although detectable at the cerebral, covert level, these changes in cerebral activity were not accompanied by obvious, overt changes in navigation efficiency. We also demonstrate a sleep-related qualitative reorganization of brain activity in showing that sleep favours the involvement of recollection processes for the retrieval of contextual memories, whereas sleep-deprivation rather implies familiarity-based responses. Additionally, our fine-grained dissection of the memory components that participate to navigation in a virtual environment indicates that sleep plays a similar promoting role in the qualitative reorganization of the brain activity that subtends consolidation of both contextual and spatial components of memory in navigation.

## Materials and Methods

### Subjects and general procedure

Twenty four right-handed volunteers (12 males, 12 females, mean age: 23.2±2.9 years) gave their written informed consent to participate in this study approved by the Ethics Committee of the Faculty of Medicine of the University of Liège. None reported any history of trauma or medical, psychiatric or sleep disorders, nor disturbances of their sleep-wake cycle during the last six weeks. Structural MRI was normal on visual inspection. They followed a constant sleep schedule (according to their own circadian habits±1h) from 3 days before the beginning of the experiment until the end of the experiment. Schedule compliance was assessed throughout using wrist actigraphy (Actiwatch, Cambridge Neuroscience).

In the sleep group (n = 12, 6 males; referred hereafter as the Regular Sleep [RS] group), subjects were allowed to sleep at home following their regular habits for the three post-learning nights. In the sleep-deprived group (n = 12, 6 males; referred as the Total Sleep Deprivation [TSD] group), subjects were kept awake in the laboratory during the first post-learning night, until 8.00 am. During this night, participants' physical activity was maintained as low as possible. Subjects remained most of the time in a seating position reading, chatting, playing quiet games or watching movies under constant supervision by the experimenters. Food intake was standardized across subjects, and luminance exposure was kept below 8 lux. At 8.00 am, subjects were allowed to leave the lab. They were instructed to follow their usual daytime activities and to abstain from napping during the day. All subjects slept normally at home on the two following (post-learning) nights. Self-report questionnaires additionally assessed sleep habits (PSQI, [57]) and circadian typology [58]. Also, sleep quality for each night from before the learning session to before the testing session was assessed using a standardized questionnaire [59]. Vigilance levels immediately before the learning and testing sessions were assessed using an adapted version of the Psychomotor Vigilance Task where simple reaction times spaced by variable intervals (2–9 s) are measured over a period of 10 min [20].

### Navigation tasks

Subjects were trained in a virtual environment previously developed and validated in our laboratory [11]–[13], [60]. The environment consists of a complex town composed of three districts, each of them containing a target location identified by a rotating medallion. The virtual town also contains 10 starting points, each located 35 virtual distance units apart from its associated target location (Figure 1). Subjects navigated within the environment in egocentric perspective at constant speed using a four-direction keypad with their right hand.

On day 1, subjects were trained outside of the scanner during a 1-hour exploration period, during which they were explicitly instructed to learn the spatial layout of streets, districts and target locations by moving freely within the environment. To promote thorough exploration of the whole town, 3 retrieval tests were administered after each 15-minute block, and subjects departed from a novel starting point the next exploration period. At the end of the training session, subjects performed 10 tests of route retrieval (Natural condition, see below) in order to assess their knowledge of the town. No fMRI data were acquired at this time. To prevent the development of anticipatory sleep strategies in subjects to be sleep-deprived, all subjects were informed of their assignment to the RS or TSD group only at the end of the learning task.

On day 4, volunteers were scanned using fMRI while performing retrieval tests. To control for potential circadian confounds, fMRI scanning took place at the same time of day than initial learning for each subject (all subjects being trained/tested between 8:00 a.m. and 6:30 p.m.). Retrieval consisted in four memory conditions (Impoverished, Recognition, Natural, and Alternate, fixed order) scanned in separate runs. These conditions were specifically designed to assess the spatial and contextual memory components engaged in spatial navigation (see [13] for a validation of these tasks). In the Natural, Impoverished and Alternate conditions, subjects had to retrieve, as fast as possible and in no more than 35 s, the route between two locations in the learned environment. In the Natural and Alternate conditions, the environment was identical to the training session. In the Alternate condition however, optimal (i.e. shorter) pathways between starting and target points were blocked by an impassable barrier to induce alternative route-finding strategies and prevent subjects navigating using a routine behavior. In the Impoverished condition, the environment was made plainly uniform by removing all wall/ground features and objects, making memory for the contextual features of the environment useless, and promoting spatial-based navigation. For each of these three tasks, the fMRI scanning session consisted of 10 blocks of tests, each lasting 35 s and alternating with 10 blocks of rest during which a black screen was displayed (random duration 10–17 s). Within the last two seconds of the rest period, the target location for the upcoming test was indicated orally through MR compatible headphones. Route retrieval performance in Impoverished and Natural conditions was computed as the remaining distance between the subject's actual location and the target to reach at the end of the test period (i.e. 35 s). In the Alternate condition where the barrier automatically made the optimal path longer than the standard 35 units in the other tests, performance was computed as the remaining distance between the subject's actual location and a virtual point located 35 units apart from the starting point on the new optimal path. In each of these three conditions, the same 10 tests were administered in a counterbalanced order between subjects.

In the Recognition condition, aimed at assessing the contextual memory component, subjects had to pay attention to the environmental features of the town while following during 35s a path signaled by colored dots displayed on the ground (Figure 1). They were instructed to determine whether and where environmental changes had been made as compared to the town explored during the learning phase. Two elements of the environment were changed in the modified paths, that did not necessitate stopping or pivoting to be easily detected. At the end of each walk, a four-choice panel composed of 3 pictures taken from the path and a white square was presented to the subjects. One of the pictures reflected the possible change made in the traveled environment. Subjects had to respond by selecting the modified image or the white square if they thought that no modification had been made (Figure 1). In this condition, 22 tests were administered in a pseudo-randomized order, alternating with short rest periods during which a black screen was displayed for 10–17 s. Behavioral performance was measured as the percentage of correct decisions. This measure was used as a performance index and correlated with the functional imaging data obtained during navigation between the starting and target points. This condition is considered here as a contextual recognition task since subjects were actively engaged in the detection of potential modifications in the learned environment.

In order to avoid as much as possible contamination from one task on the others (e.g. ongoing learning of contextual features during testing), conditions were administered in the following fixed order: Impoverished, Recognition, Natural then Alternate as in a previous validation study (see [13] for a detailed explanation of the rationale). To minimize re-learning of the contextual details of the environment or of the spatial layout of the routes, the Impoverished and Recognition conditions were administered before the Natural navigation condition. Also, some subjects followed and learned an alternate non optimal route in the Natural condition, on which they perseverated in the immediately succeeding Alternate condition, so that they never encountered the barriers on the optimal path. To take this into account, subjects' data were analyzed on the basis of the pathway they had followed in the Natural condition (whether optimal or not). In this way, we could differentiate true alternate way finding from routine strategy (see Brain Imaging and Results sections for details). At the end of the fMRI testing session, a post-experimental questionnaire was proposed in order to assess the strategies used by the subjects to perform the tasks.

Knowledge of the environment was re-tested after a delay of about 6 months. All subjects, except one performed the same tasks, in the same order, as on day 4. Only the order of the tests within each condition varied across subjects.

### fMRI data acquisition

Brain imaging data were acquired with a 3T head-only magnetic resonance (MR) scanner (Siemens, *Allegra*, Erlangen, Germany) using a blood oxygen level dependent (BOLD) sensitive single-shot echo planar (EPI) sequence (repetition time (TR) = 2130 ms; echo time (TE) = 40 ms; flip angle = 90°; field of view (FoV) = 220×220 mm^2^; matrix size = 64×64×32) covering the whole brain (128 mm high). Each functional volume consisted of 32 slices, with a thickness of 3 mm (inter-slice gap = 1 mm) and a voxel size of 3.4×3.4×3.0 mm^3^. The four initial scans of each session were discarded to control for magnetic saturation effects. A high-resolution structural MR scan was also acquired for each subject using a standard three-dimensional T1-weighted 3D MP-RAGE sequence (TR = 1960 ms; TE = 4.43 ms; flip angle = 8°; 176 slices; FoV = 230×173 mm^2^; matrix size = 256×192×176; voxel size = 0.9×0.9×0.9 mm^3^). The mean and individual MR images were used for a precise identification of loci of activation.

The virtual environment was displayed on a screen positioned at the rear of the scanner that the subject could comfortably see through a mirror mounted on the standard head coil.

### fMRI data analysis

A detailed description of functional MRI data analysis methods is provided as *Supplemental Information Text S1*. Only essential information is provided here. fMRI data were analyzed using SPM2 (http://www.fil.ion.ucl.ac.uk) implemented in MATLAB 6.1 (Mathworks). Pre-processing included realignment and adjustment for movement related effects, co-registration of functional and anatomical images, spatial normalization into standard stereotactic MNI space, and spatial smoothing using a 6 mm FWHM Gaussian kernel. Data were then analyzed using a mixed-effect model that aimed at showing stereotypical effect in the population from which the subjects are drawn [61]. This procedure was implemented in two processing steps accounting for fixed then random effects, respectively.

For each subject, changes in condition-related BOLD responses were estimated at a first, intra-individual level analysis using a general linear model at each voxel. For each experimental condition, the regressors of interest were built using boxcar functions corresponding to each 35-s block of navigation convolved with the canonical hemodynamic response function. For each test in the Impoverished, Natural and Alternate conditions, the 2-s periods during which the target to reach was orally given to the subjects were explicitly modeled. Likewise in the recognition condition, the time during which subjects were presented the choice panel and provided their response was also explicitly modeled. Furthermore, we specifically modeled subjects' behavior in the Alternate condition, in distinguishing between the *detour* and *routine* parts of their walk. These parts were individually defined with reference to each subject's behavior during the immediately preceding Natural test condition. In the special cases where a subject had followed the same, non-optimal, path as during the Natural condition (10 tests/120 in the RS group and 10/120 in TSD subjects) or when she/he never encountered the barrier (13 tests/120 in the RS group and 13/120 in TSD participants), brain activity for this test was not included in the analyses focused on a routine vs. detour distinction. Additionally, performance regressors were added to the model in order to test whether modifications of neuronal activity in navigation–related areas were linked to behavioral performance (navigation accuracy or recognition performance). This allowed computing at the within-subject level the navigation-related regional BOLD response modulated by navigation (or recognition) performance. Movement parameters were also included as covariates of no interest in the design matrix. High-pass filtering was implemented in the matrix design using a cut-off period of 128 seconds to remove low frequency drifts from the time series. Serial correlations in fMRI signal were estimated with a restricted maximum likelihood (ReML) algorithm, using an intrinsic autoregressive model during parameter estimation. Individual effects of interest (i.e., main effects of condition, direct comparisons between tasks, and intra-individual modulations by performance) were tested by linear contrasts, generating statistical parametric maps [SPM(*T*)].

These summary statistics images were then further spatially smoothed (6 mm FWHM Gaussian kernel) and entered in a second-level analysis, corresponding to a random effects (RFX) model, to evaluate differences and commonalities in brain response between the RS and TSD groups in the different conditions. The resulting set of voxel values for each contrast constituted a map of the *t* statistic [SPM(T)] thresholded at *P*<0.001 (uncorrected for multiple comparisons), corrected for multiple comparisons in a small volume of interest p^SVCcorr^<.05 for areas where a priori effects were expected (see Supplemental Table S6), otherwise corrected in the whole brain p^FWEcorr^<.05. Restricted maximum likelihood estimates of variance components were used to allow possible departure from the sphericity assumptions in RFX conjunction analyses conducted under the global null hypothesis using congruent contrasts [62]. Finally, posterior probability maps enabled conditional or Bayesian inferences about regionally specific effects, allowing us to ensure that a lack of significant statistical effect in a given contrast was not merely due to a failure to detect this effect using classical inferences [24].

## Supporting Information

Text S1 Supplemental information concerning the analysis of fMRI data and supplemental results
(0.06 MB DOC)

Table S1 Navigation performance in the four conditions according to sex and group. (1): calculated as the distance remaining to the target point; (2): calculated as the distance relative to an imaginary point located 35 units apart the starting point, on the new optimal path; *: p<0.05 as compared to men in RS group; °: p<0.05 as compared to men in TSD group ; §: p<0.06 as compared to men in RS group; •: p<0.05 as compared to women in TSD group.
(0.06 MB DOC)

Table S2 Navigation-related activity in the Natural condition, 72 h post-training. Coordinates x, y, z (mm) are given in standard stereotactic MNI space. Z = Z-statistics value. All regions listed are statistically significant at the p corrected (FWE) <0.05. For brevity, each region is listed only once; when several peaks were observed in the same region, the coordinates refer to the strongest peak. L: left; R: right.
(0.08 MB DOC)

Table S3 Navigation-related activity in the Impoverished condition, 72 h post-training. Coordinates x, y, z (mm) are given in standard stereotactic MNI space. Z = Z-statistics value. All regions listed are statistically significant at the p corrected (FWE) <0.05, excepted *: psvc(10 mm)<0.05, significant after correction in a small spherical volume (radius 10 mm) around navigation-related coordinates previously reported in the literature (see Supporting Information). For brevity, each region is listed only once; when several peaks were observed in the same region, the coordinates refer to the strongest peak. L: left; R: right.
(0.07 MB DOC)

Table S4 Navigation-related activity in the Recognition condition, 72 h post-training. Coordinates x, y, z (mm) are given in standard stereotactic MNI space. Z = Z-statistics value. All regions listed are statistically significant at the p corrected (FWE) <0.05. For brevity, each region is listed only once; when several peaks were observed in the same region, the coordinates refer to the strongest peak. L: left; R: right.
(0.08 MB DOC)

Table S5 Navigation-related activity in the Alternate condition, 72 h post-training. Coordinates x, y, z (mm) are given in standard stereotactic MNI space. All regions listed are statistically significant at the p corrected (FWE) <0.05, excepted *: psvc(10 mm)<0.05, significant after correction in a small spherical volume (radius 10 mm) around navigation-related coordinates previously reported in the literature (see Supporting Information). For brevity, each region is listed only once; when several peaks were observed in the same region, the coordinates refer to the strongest peak. L: left; R: right.
(0.11 MB DOC)

Table S6 Previously published stereotactic coordinates of navigation-related structures. 1. Maguire EA, Burgess N, Donnett JG, Frackowiak RS, Frith CD, O'Keefe J (1998a) Knowing where and getting there: a human navigation network. Science 280: 921–924. 2. Voermans NC, Petersson KM, Daudey L, Weber B, Van Spaendonck KP, Kremer HP, Fernandez G (2004) Interaction between the human hippocampus and the caudate nucleus during route recognition. Neuron 43: 427–435. 3. Iaria G, Petrides M, Dagher A, Pike B, Bohbot VD (2003) Cognitive strategies dependent on the hippocampus and caudate nucleus in human navigation: variability and change with practice. J Neurosci 23: 5945–5952. 4. Orban P, Rauchs G, Balteau E, Degueldre C, Luxen A, Maquet P, Peigneux P (2006) Sleep after spatial learning promotes covert reorganization of brain activity. Proc Natl Acad Sci U S A 103: 7124–7129. 5. Hartley T, Maguire EA, Spiers HJ, Burgess N (2003) The well-worn route and the path less travelled: distinct neural bases of route following and wayfinding in humans. Neuron 37: 877–888. 6. Bohbot VD, Iaria G, Petrides M (2004) Hippocampal function and spatial memory: evidence from functional neuroimaging in healthy participants and performance of patients with medial temporal lobe resections. Neuropsychology 18: 418–425. 7. Rauchs G, Orban P, Balteau E, Schmidt C, Degueldre C, Luxen A, Maquet P, Peigneux P (2008) Partially segregated neural networks for spatial and contextual memory in virtual navigation. Hippocampus 18: 503–518. 8. Maguire EA, Frith CD, Burgess N, Donnett JG, O'Keefe J (1998b). Knowing where things are parahippocampal involvement in encoding object locations in virtual large-scale space. J Cogn Neurosci 10: 61–76. 9. Burgess N, Maguire EA, Spiers HJ, O'Keefe J (2001) A temporoparietal and prefrontal network for retrieving the spatial context of lifelike events. Neuroimage 14: 439–453.
(0.04 MB DOC)
