# Supplementary material for: Sleep Modulates the Neural Substrates of Both Spatial and Contextual Memory Consolidation
Source: PLoS One. 2008 Aug 13;3(8):e2949. doi: 10.1371/journal.pone.0002949 (PMC2491899; doi:10.1371/journal.pone.0002949)
Supplement: Text S1 — Supplemental information concerning the analysis of fMRI data and supplemental results (0.06 MB DOC) [file pone.0002949.s001.doc]

### Sleep modulates the neural substrates of both spatial and contextual memory consolidation

**Running title**: Sleep and spatial navigation.

Géraldine Rauchs^(1,2)^, Pierre Orban^(1,3)^, Christina Schmidt^(1)^, Geneviève Albouy^(1)^, Evelyne Balteau^(1)^, Christian Degueldre^(1)^, Caroline Schnackers^(1)^, Virginie Sterpenich^(1)^, Gilberte Tinguely^(1)^, André Luxen^(1)^, Pierre Maquet^(1)^ and Philippe Peigneux^(1,4, ‡)^.

^(1)^Cyclotron Research Center, University of Liège, Belgium.

^(2)^Inserm-EPHE-Université de Caen Basse-Normandie, Unité de Recherche U923, GIP Cyceron, Caen, France

^(3)^Functional Neuroimaging Unit, University of Montréal, Canada

^(4)^ UR2N - Neuropsychology and Functional Neuroimaging Research Unit, Université Libre de Bruxelles, Belgium

**Supporting text**

**Methods**

*Detailed methods for functional MRI (fMRI) data analysis.*

Functional volumes were preprocessed and analyzed using the Statistical Parametric Mapping software SPM2 (Wellcome Department of Cognitive Neurology, London) implemented in MATLAB 6.1 (Mathworks, Sherbom, MA). Preprocessing included realignment and adjustment for movement-related effects, coregistration of functional and anatomical data, spatial normalization into standard stereotactic Montreal Neurological Institute (MNI) space, and spatial smoothing using a Gaussian kernel of 6-mm full width at half maximum (FWHM). Data were analyzed using a mixed-effects model that aimed at showing stereotypical effect in the population from which the subjects are drawn [1]. This procedure was implemented in two processing steps accounting for fixed and random effects, respectively. For each subject, a first-level intra-individual analysis estimated changes in blood-oxygen-level-dependent (BOLD) responses by a general linear model at each voxel. The regressors of interest were built using boxcars corresponding to each block of navigation convolved with the canonical hemodynamic response function. In the Impoverished, Natural and Alternate conditions, the 10 pretest 2-s periods during which subjects were orally indicated the name of the target to reach were modeled explicitly. In the Recognition condition, the time during which subjects were presented the four-choice response panel and made their response was also modeled explicitly. In the Alternate condition, the strategy used by the subjects was modeled as follows. For each test, if a subject had followed the optimal path in the preceding Natural condition, its brain activity was analyzed separately for the time spent in the detour portion of the walk, outside of its usual pathway. Otherwise, if the subject had followed the same, non-optimal, path as during the Natural condition or if she/he never encountered the barrier, brain activity during this given test was not included in the analyses.

In order to test whether modifications of neuronal activity in navigation–related areas were linked to behavioral performance (navigation accuracy for the Natural, Impoverished and Alternate conditions, percentage of correct decisions for the Recognition condition), performance regressors were added to the model. This allowed computing at the within-subject level the correlation between navigation (or recognition) performance and the navigation-related regional BOLD response. Hence, within-subjects’ correlation analyses looked for cerebral structures in which BOLD response was modulated by trial-to-trial variations in performance during tests of place finding (or during the recognition task). Movement parameters derived from realignment of the functional volumes (translations in *x*, *y*, and *z* directions and rotations around *x*, *y*, and *z* axes) were also included as covariates of no interest in the design matrix. High-pass filtering was implemented in the matrix design using a cutoff period of 128 s to remove low drift frequencies from the time series. Serial correlations were estimated with a restricted maximum likelihood (ReML) algorithm by using an intrinsic autoregressive model during parameter estimation.

The effects of interest (i.e., main effects of conditions in each group, direct comparisons between groups for the four tasks and intra-individual modulations by performance) were then tested by linear contrasts, generating statistical parametric maps [SPM(*T*)]. Since no inference was made at this fixed effects level of analysis, individual summary statistic images were thresholded at p<0.95 uncorrected, then further spatially smoothed (6 mm FWHM Gaussian kernel)*.*

These individual summary statistics images were then entered in a second-level analysis, corresponding to a random effects (RFX) model in which subjects are considered random variables, to evaluate differences and commonalities in brain response between the RS and TSD groups. The resulting set of voxel values for each contrast constituted a map of the *t* statistic [SPM(T)] thresholded at p<0.001 (uncorrected for multiple comparisons). Restricted maximum likelihood estimates of variance components were used to allow possible departure from the sphericity assumptions in RFX conjunction analyses under the global null hypothesis using congruent contrasts [2].

Finally, posterior probability maps enabled conditional or Bayesian inferences about regionally specific effects, allowing us to ensure that a lack of significant statistical effect in a given contrast was not merely due to a failure to detect this effect using classical inferences [3].

All coordinates are given as standard MNI coordinates and correspond to the maxima of the reported cluster of activation. Coordinates were labeled by using the co-planar stereotactic atlas of the human brain [4] after coordinates conversion using M. Brett’s set of linear transformations (<http://www.mrc-cbu.cam.ac.uk/Imaging/mnispace.html>) and an automatic algorithm labelling (AAL toolbox; [5]). Confirmation of precise anatomical localization was made based on individuals’ and mean structural MR images. Maps are displayed at p<0.001 uncorrected if not otherwise indicated. Functional images are displayed on the mean structural image of the subjects, normalized to the same stereotactic space.

**Results**

*Psychomotor Vigilance task (PVT)*

Reaction times were summarized by using the median, because their distribution was skewed to the left. Medians, which are approximately normally distributed, were analyzed using a repeated measures analysis of variance (ANOVA) with PVT session (before learning (day 1) *vs.* before fMRI session (day4)) as within-subject factor, and group (TSD *vs.* RS) as between-subject factor. This analysis did not revealed any significant effect of the group (F(1,22) = 0.063, p = 0.8), or of the session (F(1,22) = 0.54, p = 0.47) as well as no significant interaction between these factors (F(1,22) = 0.17, p = 0.68; RS day 1: 284.5 ± 23.8 ms, day 4: 288 ± 21.8 ms; TSD day 1: 283 ± 33.6 ms, day 4: 283.9 ± 32 ms).

A similar analysis conducted on the 10% slowest reaction times failed to reveal any significant effect of the group (F(1,22) = 0.87, p = 0.77), or of the session (F(1,22) = 2.9, p = 0.1) as well as no significant interaction between these factors (F(1,22) = 0.54, p = 0.47; RS day 1: 410.4 ± 89.3 ms, day 4: 447.6 ± 149.1 ms; TSD day 1: 443.3 ± 125.4 ms, day 4: 444.4 ± 146.2 ms). Comparable levels of vigilance between groups at the time of learning and testing indicate that sleep deprivation no longer affected vigilance after two recovery nights.

*Sleep and actigraphic data.*

As sleep semi-quantitative parameters and actigraphic data are generally not normally distributed, these data were analyzed using non-parametric tests.

Sleep duration and quality were estimated by means of questionnaires [6], the nights preceding learning and the fMRI session. Mean sleep duration was not significantly different between groups on the night before learning (RS = 467.1 ± 84.8 min, TSD = 460 ± 75.5 min; p > 0.82) and the night before fMRI session (RS = 428.8 ± 58.9 min, TSD = 459.2 ± 83.9 min; p > 0.34). Within each group, duration of each night did not differ significantly (RS group: p > 0.38; TSD group: p > 0.75). Likewise, a similar analysis conducted on subjective sleep quality, assessed by means of a 6-point scale, failed to evidence any between-group differences. Subjective sleep quality was equivalent between groups for each night (night before learning: RS = 4.75 ± 1.1, TSD = 5.2 ± 0.8; p > 0.4; night before fMRI session: RS = 5.3 ± 0.8, TSD = 5 ± 0.8; p > 0.46). Finally, within group comparisons did not reveal any significant difference in sleep quality between both nights (RS group: p > 0.07; TSD group: p > 0.62).

Actigraphic data (mean wrist movements activity, in arbitrary units) of post-training nights and days were distinguished on the basis of the timetable provided by the subjects in their sleep questionnaire. Data of the three post-learning nights were then compared using Mann-Whitney U tests. As expected, activity was larger in the TSD than in the RS group during the first night (RS = 26.3 ± 12.2 arbitrary units, TSD = 276.7 ± 93.7 units, p<0.001). During the second and third nights, activity in TSD subjects was not different that in RS subjects (Night 2: RS = 28 ± 19.1 units, TSD = 36 ± 30.9 units, p=0.64; Night 3: RS = 26.3 ± 15.8 units, TSD = 39.4 ± 21.9 units, p=0.22), suggesting together with the results of the psychomotor vigilance task that testing subjects two nights after a sleep deprivation is a time interval sufficient to dissipate most of the side-effects of sleep deprivation (e.g. tiredness, low vigilance). In addition, a similar analysis carried out on actigraphic data gathered during the second day of the experiment (i.e., following the sleep deprivation for subjects in the TSD group) failed to reveal any between group differences (p > 0.44).

*Post-experimental questionnaire*

This questionnaire was proposed to get information about the strategies used by the subjects to perform the various tasks. In the RS group, all subjects claimed using elements of the scenery to find their way in the environment. 7 out of the 12 subjects also used the shape of rooms and hallways to get one’s bearings, and 2 out of 12 subjects memorized sequences of left/right turns. In the TSD group, things were broadly the same: all subjects but one reported using elements of the scenery. The only subject that did not use this strategy rather tried to create a mental map of the town. 8 subjects out of 12 reported to have relied on a spatial strategy (learning the shapes of rooms) and 2 memorized left/right sequences of turns. These data indicate that there is no obvious difference in the way RS and TSD participants have solved the tasks.

**References**

[1] Penny W, Holmes A (2003) Random-effect analysis. In: Frackowiak RJS, Friston KJ, Frith C, Dolan R, Price CJ, Zeki S, Ashburner J, editors. Human Brain Function. 2nd edition. London: Academic Press. pp. 843-850.

[2] Friston KJ, Glaser DE, Henson RN, Kiebel S, Phillips C, et al (2002) Classical and Bayesian inference in neuroimaging: applications. Neuroimage 16: 484–512.

[3] Friston KJ, Penny W (2003) Posterior probability maps and SPMs. Neuroimage 19:1240-1249.

[4] Talairach J, Tournoux P (1988) Co-Planar Stereotaxic Atlas of the Human Brain: Three-Dimensional Proportional System. Georg Thieme, Stuttgart, Germany. 122 p.

[5] Tzourio-Mazoyer N, Landeau B, Papathanassiou D, Crivello F, Etard O, et al (2002) Automated anatomical labeling of activations in SPM using a macroscopic anatomical parcellation of the MNI MRI single-subject brain. Neuroimage 15: 273-289.

[6] Ellis BW, Johns MW, Lancaster R, Raptopoulos P, Angelopoulos N, et al (1981). The St. Mary's Hospital sleep questionnaire: a study of reliability. Sleep 4: 93-97.

**Table legends.**

**Table S1: Navigation performance in the four conditions according to sex and group.**

(1): calculated as the distance remaining to the target point.

(2): calculated as the distance relative to an imaginary point located 35 units apart the starting point, on the new optimal path.

*: p<0.05 as compared to men in RS group.

°: p<0.05 as compared to men in TSD group

§: p<0.06 as compared to men in RS group

•: p<0.05 as compared to women in TSD group.

**Table S2: Navigation-related activity in the Natural condition, 72h post-training.**

Coordinates x, y, z (mm) are given in standard stereotactic MNI space. Z = Z-statistics value. All regions listed are statistically significant at the p corrected (FWE) <0.05. For brevity, each region is listed only once; when several peaks were observed in the same region, the coordinates refer to the strongest peak. L: left; R: right.

**Table S3: Navigation-related activity in the Impoverished condition, 72h post-training.**

Coordinates x, y, z (mm) are given in standard stereotactic MNI space. Z = Z-statistics value. All regions listed are statistically significant at the p corrected (FWE) <0.05, excepted *: psvc(10 mm)<0.05, significant after correction in a small spherical volume (radius 10 mm) around navigation-related coordinates previously reported in the literature (see Supporting Information). For brevity, each region is listed only once; when several peaks were observed in the same region, the coordinates refer to the strongest peak. L: left; R: right.

**Table S4: Navigation-related activity in the Recognition condition, 72h post-training.**

Coordinates x, y, z (mm) are given in standard stereotactic MNI space. Z = Z-statistics value. All regions listed are statistically significant at the p corrected (FWE) <0.05. For brevity, each region is listed only once; when several peaks were observed in the same region, the coordinates refer to the strongest peak. L: left; R: right.

**Table S5:** **Navigation-related activity in the Alternate condition, 72h post-training.**

Coordinates x, y, z (mm) are given in standard stereotactic MNI space. All regions listed are statistically significant at the p corrected (FWE) <0.05, excepted *: psvc(10 mm)<0.05, significant after correction in a small spherical volume (radius 10 mm) around navigation-related coordinates previously reported in the literature (see Supporting Information). For brevity, each region is listed only once; when several peaks were observed in the same region, the coordinates refer to the strongest peak. L: left; R: right.

**Table S6: Previously published stereotactic coordinates of navigation-related structures.**

1. Maguire EA, Burgess N, Donnett JG, Frackowiak RS, Frith CD, O'Keefe J (1998a) Knowing where and getting there: a human navigation network. Science 280: 921-924.

2. Voermans NC, Petersson KM, Daudey L, Weber B, Van Spaendonck KP, Kremer HP, Fernandez G (2004) Interaction between the human hippocampus and the caudate nucleus during route recognition. Neuron 43: 427-435.

3. Iaria G, Petrides M, Dagher A, Pike B, Bohbot VD (2003) Cognitive strategies dependent on the hippocampus and caudate nucleus in human navigation: variability and change with practice. J Neurosci 23: 5945-5952.

4. Orban P, Rauchs G, Balteau E, Degueldre C, Luxen A, Maquet P, Peigneux P (2006) Sleep after spatial learning promotes covert reorganization of brain activity. Proc Natl Acad Sci U S A 103: 7124-7129.

5. Hartley T, Maguire EA, Spiers HJ, Burgess N (2003) The well-worn route and the path less travelled: distinct neural bases of route following and wayfinding in humans. Neuron 37: 877-888.

6. Bohbot VD, Iaria G, Petrides M (2004) Hippocampal function and spatial memory: evidence from functional neuroimaging in healthy participants and performance of patients with medial temporal lobe resections. Neuropsychology 18: 418-425.

7. Rauchs G, Orban P, Balteau E, Schmidt C, Degueldre C, Luxen A, Maquet P, Peigneux P (2008) Partially segregated neural networks for spatial and contextual memory in virtual navigation. Hippocampus 18: 503-518.

8. Maguire EA, Frith CD, Burgess N, Donnett JG, O'Keefe J (1998b). Knowing where things are parahippocampal involvement in encoding object locations in virtual large-scale space. J Cogn Neurosci 10: 61-76.

9. Burgess N, Maguire EA, Spiers HJ, O'Keefe J (2001) A temporoparietal and prefrontal network for retrieving the spatial context of lifelike events. Neuroimage 14: 439-453.
