# Supplementary material for: Sleep Modulates the Neural Substrates of Both Spatial and Contextual Memory Consolidation
Source: PLoS One. 2008 Aug 13;3(8):e2949. doi: 10.1371/journal.pone.0002949 (PMC2491899; doi:10.1371/journal.pone.0002949)
Supplement: Table S1 — Navigation performance in the four conditions according to sex and group. (1): calculated as the distance remaining to the target point; (2): calculated as the distance relative to an imaginary point located 35 units apart the starting point, on the new optimal path; *: p<0.05 as compared to men in RS group; °: p<0.05 as compared to men in TSD group ; §: p<0.06 as compared to men in RS group; •: p<0.05 as compared to women in TSD group. (0.06 MB DOC) [file pone.0002949.s002.doc]

**Table S1: Navigation performance in the four conditions according to sex and group.**

|  |  | **Group** | | | |
| --- | --- | --- | --- | --- | --- |
|  |  | **RS** | | **TSD** | |
|  |  | **Men** | **Women** | **Men** | **Women** |
| Performance at the end of the training session | | 6.7 ± 7 | 15.9 ± 6.3 * | 8.3 ± 3.9 | 16.6 ± 8.8 * |
|  |  |  |  |  |  |
| *Natural condition* | |  |  |  |  |
|  | mean performance | 7.8 ± 5.3 | 15.4 ± 9 §° | 5.9 ± 4.3 | 18.1 ± 7.6 *° |
|  | mean effective speed | 1.2 ± 0 | 1.1 ± 0.1 ° | 1.3 ± 0.2 | 1.2 ± 0.1 |
|  | time spent hesitating | 13.5 ± 11.1 | 33.2 ± 11.7 *° ^•^ | 18.3 ± 11 | 12.3 ± 13.9 |
|  | mean number of hesitations at cross-roads | 3.8 ± 3.2 | 6.8 ± 3.7 | 3.3 ± 2.3 | 3 ± 3.3 |
|  | mean number of dead ends visited | 1.5 ± 1.4 | 2.5 ± 1.9 | 2.2 ± 2 | 0.8 ± 0.8 |
|  |  |  |  |  |  |
| *Impoverished condition* | |  |  |  |  |
|  | mean performance | 20.6 ± 6.2 | 29.1 ± 11.1 | 23.9 ± 10.5 | 24.5 ± 8.7 |
|  | mean effective speed | 1.1 ± 0 | 0.9 ± 0.1 * | 1.1 ± 0.1 | 1 ± 0.1 |
|  | time spent hesitating | 28.3 ± 9.1 | 53.8 ± 15.2 * | 36.5 ± 21.5 | 33.7 ± 23.8 |
|  | mean number of hesitations at cross-roads | 6.8 ± 2.1 | 10.2 ± 2.6 | 7.2 ± 3.7 | 7.2 ± 5 |
|  | mean number of dead ends visited | 3.3 ± 1 | 3.3 ± 2.3 | 3.3 ± 1.9 | 4.2 ± 2 |
|  |  |  |  |  |  |
| *Alternate condition* | |  |  |  |  |
|  | mean performance (1) | 18.2 ± 5.8 | 26.2 ± 8.3 | 19.5 ± 5.1 | 24.1 ± 7.4 |
|  | mean performance (2) | 5.9 ± 5.6 | 12 ± 9.3 | 7.9 ± 3.9 | 10.3 ± 5.9 |
|  | mean effective speed | 1.2 ± 0 | 1 ± 0.1 | 1.2 ± 0.2 | 1.1 ± 0.2 |
|  | time spent hesitating | 6 ± 2.4 | 20 ± 8.3 *° ^•^ | 8.5 ± 7.7 | 6 ± 2.8 |
|  | mean number of hesitations at cross-roads | 0.7 ± 0.5 | 3.2 ± 1.8 * | 1.8 ± 1.3 | 1.5 ± 1.8 |
|  | mean number of dead ends visited | 0.7 ± 1.2 | 0.7 ± 0.5 | 1.2 ± 1.5 | 1.7 ± 1.9 |
|  | mean time spent having a detour strategy | 20.8 ± 2 | 17 ± 5.8 | 17.3 ± 3.1 | 17.6 ± 2.5 |
|  | mean time spent having a routine strategy | 9.8 ± 0.6 | 10 ± 3.3 | 10.3 ± 2.4 | 11.5 ± 1.4 |
|  | mean time spent being lost | 4.1 ± 2.8 | 4.9 ± 2.5 | 7.4 ± 4.7 | 5.5 ± 2.6 |
|  |  |  |  |  |  |
| *Recognition task* | |  |  |  |  |
|  | mean percentage of correct recognitions | 70.3 ± 11.3 | 58.1 ± 23.6 | 56.7 ± 12.6 | 68.6 ± 17.3 |
