# Supplementary material for: Sleep Modulates the Neural Substrates of Both Spatial and Contextual Memory Consolidation
Source: PLoS One. 2008 Aug 13;3(8):e2949. doi: 10.1371/journal.pone.0002949 (PMC2491899; doi:10.1371/journal.pone.0002949)
Supplement: Table S2 — Navigation-related activity in the Natural condition, 72 h post-training. Coordinates x, y, z (mm) are given in standard stereotactic MNI space. Z = Z-statistics value. All regions listed are statistically significant at the p corrected (FWE) <0.05. For brevity, each region is listed only once; when several peaks were observed in the same region, the coordinates refer to the strongest peak. L: left; R: right. (0.08 MB DOC) [file pone.0002949.s003.doc]

**Table S2: Navigation-related activity in the Natural condition, 72h post-training.**

| **Region** | **RS** | | | | **TSD** | | | |
| --- | --- | --- | --- | --- | --- | --- | --- | --- |
|  | **x** | **y** | **z** | **Z** | **x** | **y** | **z** | **Z** |
| *Frontal areas* |  |  |  |  |  |  |  |  |
| L superior frontal gyrus | -32 | -12 | 58 | 6.38 | -18 | -8 | 76 | 5.03 |
| R superior frontal gyrus | 30 | 0 | 56 | 5.98 | 28 | 6 | 64 | 5.08 |
| R inferior frontal gyrus | 34 | 24 | -8 | 5.26 |  |  |  |  |
| R inferior frontal operculum | 62 | 14 | 22 | 5.16 |  |  |  |  |
| R precentral gyrus | 48 | 8 | 34 | 5.15 |  |  |  |  |
| L precentral gyrus | -32 | -2 | 64 | 6.95 | -32 | -2 | 64 | 5.56 |
| L supplementary motor area | -4 | 0 | 52 | 5.55 | -12 | -2 | 76 | 5.00 |
| R supplementary motor area | 8 | 18 | 50 | 5.15 |  |  |  |  |
| L middle cingulate gyrus | -10 | -16 | 44 | 6.41 | -10 | -18 | 44 | 5.70 |
| R middle cingulate gyrus | 16 | -18 | 42 | 5.91 |  |  |  |  |
|  |  |  |  |  |  |  |  |  |
| *Parietal areas* |  |  |  |  |  |  |  |  |
| L insula | -34 | 22 | -8 | 5.18 |  |  |  |  |
| L postcentral gyrus | -38 | -24 | 56 | 5.90 |  |  |  |  |
| L superior parietal gyrus |  |  |  |  | -14 | -76 | 54 | 5.84 |
| L inferior parietal gyrus | -50 | -26 | 38 | 5.14 |  |  |  |  |
| R precuneus |  |  |  |  | 8 | -46 | 66 | 5.17 |
| L precuneus | -24 | -64 | 26 | 4.95 | -14 | -58 | 16 | 6.03 |
| R cuneus |  |  |  |  | 22 | -58 | 20 | 6.58 |
| R retrosplenial cortex |  |  |  |  | 10 | -54 | 6 | 5.12 |
|  |  |  |  |  |  |  |  |  |
| *Occipital areas* |  |  |  |  |  |  |  |  |
| L fusiform gyrus | -30 | -56 | -12 | 7.53 | -30 | -54 | -12 | 6.94 |
| R lingual gyrus | 10 | -28 | -6 | 5.82 | 10 | -28 | -6 | 5.62 |
| R middle occipital gyrus | 34 | -90 | 8 | 7.50 | 34 | -90 | 8 | 7.11 |
| L inferior occipital gyrus | -40 | -82 | -10 | 7.43 |  |  |  |  |
| L calcarine region | -16 | -60 | 16 | 5.93 |  |  |  |  |
|  |  |  |  |  |  |  |  |  |
| *Other structures* |  |  |  |  |  |  |  |  |
| L thalamus | -14 | -22 | 8 | 4.97 |  |  |  |  |
| L hippocampus | -22 | -28 | -6 | 6.03 | -22 | -28 | -6 | 5.73 |
| R hippocampus | 24 | -28 | -2 | 5.77 |  |  |  |  |
| L cerebelum | -38 | -46 | -36 | 5.30 | -22 | -82 | -20 | 7.25 |
| Vermis | -2 | -60 | -42 | 5.20 |  |  |  |  |
