# Supplementary material for: Sleep Modulates the Neural Substrates of Both Spatial and Contextual Memory Consolidation
Source: PLoS One. 2008 Aug 13;3(8):e2949. doi: 10.1371/journal.pone.0002949 (PMC2491899; doi:10.1371/journal.pone.0002949)
Supplement: Table S3 — Navigation-related activity in the Impoverished condition, 72 h post-training. Coordinates x, y, z (mm) are given in standard stereotactic MNI space. Z = Z-statistics value. All regions listed are statistically significant at the p corrected (FWE) <0.05, excepted *: psvc(10 mm)<0.05, significant after correction in a small spherical volume (radius 10 mm) around navigation-related coordinates previously reported in the literature (see Supporting Information). For brevity, each region is listed only once; when several peaks were observed in the same region, the coordinates refer to the strongest peak. L: left; R: right. (0.07 MB DOC) [file pone.0002949.s004.doc]

**Table S3: Navigation-related activity in the Impoverished condition, 72h post-training.**

| **Region** | **RS** | | | | **TSD** | | | |
| --- | --- | --- | --- | --- | --- | --- | --- | --- |
|  | **x** | **y** | **z** | **Z** | **x** | **y** | **z** | **Z** |
| *Frontal areas* |  |  |  |  |  |  |  |  |
| R middle frontal gyrus | 30 | 0 | 56 | 5.46 |  |  |  |  |
| R superior frontal gyrus | 24 | -2 | 62 | 5.36 |  |  |  |  |
| L precentral gyrus | -28 | -12 | 50 | 5.41 |  |  |  |  |
| L middle cingulate gyrus | -12 | -16 | 46 | 5.19 |  |  |  |  |
|  |  |  |  |  |  |  |  |  |
| *Parietal areas* |  |  |  |  |  |  |  |  |
| L superior parietal gyrus | -26 | -56 | 62 | 5.59 |  |  |  |  |
| R superior parietal gyrus |  |  |  |  | 18 | -56 | 58 | 5.30 |
| R precuneus |  |  |  |  | 10 | -74 | 60 | 5.56 |
| R cuneus | 22 | -60 | 20 | 5.87 |  |  |  |  |
|  |  |  |  |  |  |  |  |  |
| *Temporal areas* |  |  |  |  |  |  |  |  |
| L parahippocampal gyrus | -20 | -36 | -12 | 4.03 | -18 | -34 | -14 | 3.12 |
| R parahippocampal gyrus | 24 | -36 | -10 | 3.26 |  |  |  |  |
| R inferior temporal gyrus |  |  |  |  | 52 | -72 | -6 | 5.52 |
| R hippocampus |  |  |  |  | 24 | -36 | -10 | 3.20 |
|  |  |  |  |  |  |  |  |  |
| *Occipital areas* |  |  |  |  |  |  |  |  |
| L middle occipital gyrus | -26 | -86 | 22 | 6.86 | -26 | -84 | 22 | 5.97 |
| R middle occipital gyrus | 34 | -90 | 8 | 6.61 | 34 | -78 | 30 | 5.95 |
| L fusiform gyrus | -30 | -40 | -14 | 3.81 | -22 | -38 | -20 | 3.43 |
| R lingual gyrus |  |  |  |  | 8 | -94 | -10 | 5.60 |
| R calcarine |  |  |  |  | 24 | -96 | 2 | 4.92 |
|  |  |  |  |  |  |  |  |  |
| *Other structures* |  |  |  |  |  |  |  |  |
| L cerebelum | -10 | -80 | -20 | 6.55 | -24 | -80 | -22 | 5.96 |
| R cerebelum |  |  |  |  | 30 | -51 | -24 | 6.44 |
