# Supplementary material for: Sleep Modulates the Neural Substrates of Both Spatial and Contextual Memory Consolidation
Source: PLoS One. 2008 Aug 13;3(8):e2949. doi: 10.1371/journal.pone.0002949 (PMC2491899; doi:10.1371/journal.pone.0002949)
Supplement: Table S4 — Navigation-related activity in the Recognition condition, 72 h post-training. Coordinates x, y, z (mm) are given in standard stereotactic MNI space. Z = Z-statistics value. All regions listed are statistically significant at the p corrected (FWE) <0.05. For brevity, each region is listed only once; when several peaks were observed in the same region, the coordinates refer to the strongest peak. L: left; R: right. (0.08 MB DOC) [file pone.0002949.s005.doc]

**Table S4: Navigation-related activity in the Recognition condition, 72h post-training.**

| **Region** | | **RS** | | | | **TSD** | | | |
| --- | --- | --- | --- | --- | --- | --- | --- | --- | --- |
|  |  | **x** | **y** | **z** | **Z** | **x** | **y** | **z** | **Z** |
| *Frontal areas* | |  |  |  |  |  |  |  |  |
| R precentral gyrus | | 28 | -2 | 56 | 6.45 |  |  |  |  |
| R inferior frontal operculum | | 64 | 12 | 20 | 6.09 |  |  |  |  |
| L superior frontal gyrus | |  |  |  |  | -22 | -2 | 64 | 5.61 |
| R superior frontal gyrus | | 18 | -6 | 68 | 6.03 | 4 | 14 | 50 | 5.15 |
| R middle frontal gyrus | |  |  |  |  | 28 | 0 | 56 | 5.24 |
| R supplementary motor area | | 6 | 14 | 50 | 6.32 | 14 | -2 | 68 | 5.15 |
| L supplementary motor area | | -6 | 2 | 50 | 5.38 |  |  |  |  |
| L precentral gyrus | | -28 | -4 | 62 | 6.25 | -40 | -4 | 54 | 5.60 |
| L middle cingulate gyrus | | -12 | -18 | 46 | 5.18 |  |  |  |  |
| R middle cingulate gyrus | | 16 | -20 | 46 | 4.99 |  |  |  |  |
|  |  |  |  |  |  |  |  |  |  |
| *Parietal areas* | |  |  |  |  |  |  |  |  |
| L postcentral gyrus | | -42 | -16 | 52 | 5.46 |  |  |  |  |
| R superior parietal gyrus | |  |  |  |  | 20 | -62 | 70 | 6.00 |
| R inferior parietal gyrus | | 46 | -34 | 46 | 5.22 |  |  |  |  |
| R precuneus |  |  |  |  |  | 6 | -64 | 66 | 6.24 |
|  |  |  |  |  |  |  |  |  |  |
| *Temporal areas* | |  |  |  |  |  |  |  |  |
| L temporal pole | | -30 | 18 | -30 | 5.67 |  |  |  |  |
|  |  |  |  |  |  |  |  |  |  |
| *Occipital areas* | |  |  |  |  |  |  |  |  |
| L superior occipital gyrus | |  |  |  |  | -28 | -72 | 32 | 5.11 |
| L middle occipital gyrus | | 36 | -90 | 10 | >8 | -28 | -94 | 18 | 6.65 |
| R middle occipital gyrus | |  |  |  |  | 36 | -90 | 10 | 7.42 |
| L inferior occipital gyrus | | -40 | -78 | -10 | 7.32 |  |  |  | 6.81 |
| R inferior occipital gyrus | | 46 | -68 | -14 | 7.30 |  |  |  |  |
| L lingual gyrus | |  |  |  |  | -6 | -94 | -10 | 6.81 |
|  |  |  |  |  |  |  |  |  |  |
| *Other structures* | |  |  |  |  |  |  |  |  |
| R thalamus |  | 26 | -26 | -4 | 5.97 | 24 | -26 | -2 | 5.40 |
| L thalamus |  | -24 | -26 | -6 | 5.73 | -22 | -28 | -2 | 5.01 |
| Vermis |  | 2 | -72 | -38 | 5.13 | 6 | -54 | 2 | 5.26 |
| Cerebelum |  | 0 | -58 | -46 | 5.00 | -42 | -46 | -42 | 5.10 |
