# Supplementary material for: Sleep Modulates the Neural Substrates of Both Spatial and Contextual Memory Consolidation
Source: PLoS One. 2008 Aug 13;3(8):e2949. doi: 10.1371/journal.pone.0002949 (PMC2491899; doi:10.1371/journal.pone.0002949)
Supplement: Table S5 — Navigation-related activity in the Alternate condition, 72 h post-training. Coordinates x, y, z (mm) are given in standard stereotactic MNI space. All regions listed are statistically significant at the p corrected (FWE) <0.05, excepted *: psvc(10 mm)<0.05, significant after correction in a small spherical volume (radius 10 mm) around navigation-related coordinates previously reported in the literature (see Supporting Information). For brevity, each region is listed only once; when several peaks were observed in the same region, the coordinates refer to the strongest peak. L: left; R: right. (0.11 MB DOC) [file pone.0002949.s006.doc]

**Table S5:** **Navigation-related activity in the Alternate condition, 72h post-training.**

| **Region** | | **RS** | | | | **TSD** | | | |
| --- | --- | --- | --- | --- | --- | --- | --- | --- | --- |
|  |  | **x** | **y** | **z** | **Z** | **x** | **y** | **z** | **Z score** |
| *Activations during the Detour part of the route* | | | |  |  |  |  |  |  |
|  |  |  |  |  |  |  |  |  |  |
|  | R superior frontal gyrus | 26 | 0 | 56 | 5.85 | 22 | 4 | 58 | 5.15 |
|  | L superior frontal gyrus |  |  |  |  | -20 | 0 | 62 | 5.47 |
|  | L precentral gyrus | -26 | -2 | 60 | 5.62 |  |  |  |  |
|  | L middle cingulate gyrus | -12 | -16 | 44 | 5.31 | -14 | -16 | 44 | 5.14 |
|  | L superior parietal gyrus |  |  |  |  | -14 | -72 | 52 | 5.09 |
|  | R caudate nucleus (head) | 6 | 18 | 4 | 3.25* |  |  |  |  |
|  | R caudate nucleus (body) |  |  |  |  | 2 | 16 | 8 | 3.44* |
|  | L pallidum |  |  |  |  | -16 | -8 | -6 | 5 |
|  | R thalamus | 22 | -28 | -2 | 5.94 |  |  |  |  |
|  | R hippocampus | 24 | -28 | -2 | 5.85 |  |  |  |  |
|  | L hippocampus | -20 | -30 | -4 | 5.19 |  |  |  |  |
|  | R parahippocampal gyrus | 28 | -44 | -14 | 5.90* | 30 | -44 | -14 | 5.34* |
|  | L fusiform gyrus | -30 | -54 | -12 | 6.39* | -28 | -52 | -12 | 5.69* |
|  | R inferior temporal gyrus |  |  |  |  | 46 | -64 | -4 | 5.33 |
|  | R inferior occipital gyrus | 46 | -72 | -16 | 5.39 |  |  |  |  |
|  | L precuneus | -14 | -58 | 16 | 6.02 | -6 | -72 | 62 | 4.86 |
|  | R cuneus | 22 | -66 | 30 | 6.95 |  |  |  |  |
|  | L cuneus |  |  |  |  | -14 | -58 | 18 | 5.74 |
|  | L calcarine region |  |  |  |  | -6 | -94 | -10 | 6.89 |
|  | R middle occipital gyrus | 34 | -90 | 10 | 7.35 | 34 | -92 | 12 | 6.49 |
|  | L cerebelum | -12 | -82 | -18 | 7.20 | -24 | -82 | -20 | 6.43 |
|  | R cerebelum |  |  |  |  | 38 | -48 | -30 | 4.98 |
|  | Vermis | 2 | -62 | -42 | 4.89 |  |  |  |  |
|  |  |  |  |  |  |  |  |  |  |
| *Activations during the Routine part of the route* | | | |  |  |  |  |  |  |
|  |  |  |  |  |  |  |  |  |  |
|  | R superior frontal gyrus | 28 | 0 | 56 | 5.72 | 24 | 6 | 58 | 5.20 |
|  | L superior frontal gyrus | -26 | -2 | 62 | 6.10 | -24 | -2 | 62 | 5.88 |
|  | R supplementary motor area | 10 | 16 | 50 | 5.06 |  |  |  |  |
|  | R insula | 32 | 24 | -2 | 5.58 | 34 | 26 | -2 | 5.00 |
|  | R superior parietal gyrus |  |  |  |  | 20 | -60 | 66 | 5.82 |
|  | L superior parietal gyrus |  |  |  |  | -14 | -72 | 52 | 5.24 |
|  | R caudate nucleus (head) |  |  |  |  | 4 | 14 | 4 | 3.22* |
|  | R caudate nucleus (body) |  |  |  |  | 8 | 10 | 10 | 3.55* |
|  | R thalamus | 24 | -28 | -4 | 5.42 | 22 | -26 | -4 | 5.78 |
|  | L thalamus | -20 | -28 | -4 | 5.01 |  |  |  |  |
|  | L parahippocampal gyrus | -30 | -54 | -10 | 6.31* | -28 | -54 | -10 | 5.5* |
|  | R fusiform gyrus |  |  |  |  | 30 | -50 | -14 | 5.13 |
|  | R inferior temporal gyrus |  |  |  |  | 48 | -70 | 2 | 5.20 |
|  | R precuneus |  |  |  |  | 18 | -58 | 20 | 5.97 |
|  | L precuneus |  |  |  |  | -12 | -56 | 18 | 5.77 |
|  | R retrosplenial cortex |  |  |  |  | 8 | -54 | 6 | 5.00 |
|  | L calcarine region | -6 | -94 | -10 | 7.03 |  |  |  |  |
|  | R middle occipital gyrus | 34 | -92 | 12 | 7.34 | 34 | -92 | 12 | 6.59 |
|  | L middle occipital gyrus |  |  |  |  | -6 | -94 | -10 | 6.95 |
|  | R inferior occipital gyrus | 46 | -72 | -14 | 5.36 |  |  |  |  |
|  | L cerebelum | -14 | -82 | -18 | 7.25 | -14 | -84 | -18 | 6.70 |
|  | Vermis | 0 | -60 | -42 | 5.11 | 0 | -62 | -40 | 4.91 |
