# Supplementary material for: Sleep Modulates the Neural Substrates of Both Spatial and Contextual Memory Consolidation
Source: PLoS One. 2008 Aug 13;3(8):e2949. doi: 10.1371/journal.pone.0002949 (PMC2491899; doi:10.1371/journal.pone.0002949)
Supplement: Table S6 — Previously published stereotactic coordinates of navigation-related structures. 1. Maguire EA, Burgess N, Donnett JG, Frackowiak RS, Frith CD, O'Keefe J (1998a) Knowing where and getting there: a human navigation network. Science 280: 921–924. 2. Voermans NC, Petersson KM, Daudey L, Weber B, Van Spaendonck KP, Kremer HP, Fernandez G (2004) Interaction between the human hippocampus and the caudate nucleus during route recognition. Neuron 43: 427–435. 3. Iaria G, Petrides M, Dagher A, Pike B, Bohbot VD (2003) Cognitive strategies dependent on the hippocampus and caudate nucleus in human navigation: variability and change with practice. J Neurosci 23: 5945–5952. 4. Orban P, Rauchs G, Balteau E, Degueldre C, Luxen A, Maquet P, Peigneux P (2006) Sleep after spatial learning promotes covert reorganization of brain activity. Proc Natl Acad Sci U S A 103: 7124–7129. 5. Hartley T, Maguire EA, Spiers HJ, Burgess N (2003) The well-worn route and the path less travelled: distinct neural bases of route following and wayfinding in humans. Neuron 37: 877–888. 6. Bohbot VD, Iaria G, Petrides M (2004) Hippocampal function and spatial memory: evidence from functional neuroimaging in healthy participants and performance of patients with medial temporal lobe resections. Neuropsychology 18: 418–425. 7. Rauchs G, Orban P, Balteau E, Schmidt C, Degueldre C, Luxen A, Maquet P, Peigneux P (2008) Partially segregated neural networks for spatial and contextual memory in virtual navigation. Hippocampus 18: 503–518. 8. Maguire EA, Frith CD, Burgess N, Donnett JG, O'Keefe J (1998b). Knowing where things are parahippocampal involvement in encoding object locations in virtual large-scale space. J Cogn Neurosci 10: 61–76. 9. Burgess N, Maguire EA, Spiers HJ, O'Keefe J (2001) A temporoparietal and prefrontal network for retrieving the spatial context of lifelike events. Neuroimage 14: 439–453. (0.04 MB DOC) [file pone.0002949.s007.doc]

**Table S6: Previously published stereotactic coordinates of navigation-related structures.**

| Brain area | Stereotactic coordinates (Montreal Neurological Institute) | | | Reference |
| --- | --- | --- | --- | --- |
|
| Caudate nucleus | 10 | 14 | -4 | 1 |
|  | 14 | 10 | 18 | 2 |
|  | 6 | 14 | 2 | 2 |
|  | -20 | -2 | -4 | 3 |
|  | 14 | 8 | 18 | 4 |
|  | 8 | 22 | 4 | 4 |
|  |  |  |  |  |
| Hippocampus | 30 | -33 | 0 | 5 |
|  | 32 | -34 | 8 | 3 |
|  | 22 | -16 | -14 | 3,6 |
|  | 16 | -14 | 20 | 2 |
|  | -22 | -28 | -12 | 2 |
|  | -26 | -34 | -6 | 3 |
|  | 22 | 12 | 22 | 4 |
|  | 24 | -28 | -4 | 7 |
|  | -22 | -28 | -8 | 7 |
|  |  |  |  |  |
| Parahippocampal gyrus | 22 | -40 | -8 | 8 |
|  | 22 | -30 | -20 | 8 |
|  | 24 | -33 | -18 | 9 |
|  | 30 | -45 | -12 | 9 |
|  | -24 | -42 | -15 | 9 |
